# Mitogen activated protein kinase (MAPK)-regulated genes with predicted signal peptides function in the *Glycine max* defense response to the root pathogenic nematode *Heterodera glycines*

**DOI:** 10.1371/journal.pone.0241678

**Published:** 2020-11-04

**Authors:** Prakash M. Niraula, Keshav Sharma, Brant T. McNeece, Hallie A. Troell, Omar Darwish, Nadim W. Alkharouf, Katherine S. Lawrence, Vincent P. Klink

**Affiliations:** 1 Department of Biological Sciences, Mississippi State University, Mississippi State, MS, United States of America; 2 Department of Mathematics and Computer Science, Texas Women’s University, Denton, TX, United States of America; 3 Department of Computer and Information Sciences, Towson University, Towson, MD, United States of America; 4 Department of Entomology and Plant Pathology, Auburn University, Auburn, AL, United States of America; 5 Department of Biochemistry, Molecular Biology, Entomology and Plant Pathology, Mississippi State University, Mississippi State, MS, United States of America; 6 Center for Computational Sciences High Performance Computing Collaboratory, Mississippi State University, Starkville, MS, United States of America; Southern Crop Protection and Food Research Centre, CANADA

## Abstract

*Glycine max* has 32 mitogen activated protein kinases (MAPKs), nine of them exhibiting defense functions (defense MAPKs) to the plant parasitic nematode *Heterodera glycines*. RNA seq analyses of transgenic *G*. *max* lines overexpressing (OE) each defense MAPK has led to the identification of 309 genes that are increased in their relative transcript abundance by all 9 defense MAPKs. Here, 71 of those genes are shown to also have measurable amounts of transcript in *H*. *glycines*-induced nurse cells (syncytia) produced in the root that are undergoing a defense response. The 71 genes have been grouped into 7 types, based on their expression profile. Among the 71 genes are 8 putatively-secreted proteins that include a galactose mutarotase-like protein, pollen Ole e 1 allergen and extensin protein, endomembrane protein 70 protein, O-glycosyl hydrolase 17 protein, glycosyl hydrolase 32 protein, FASCICLIN-like arabinogalactan protein 17 precursor, secreted peroxidase and a pathogenesis-related thaumatin protein. Functional transgenic analyses of all 8 of these candidate defense genes that employ their overexpression and RNA interference (RNAi) demonstrate they have a role in defense. Overexpression experiments that increase the relative transcript abundance of the candidate defense gene reduces the ability that the plant parasitic nematode *Heterodera glycines* has in completing its life cycle while, in contrast, RNAi of these genes leads to an increase in parasitism. The results provide a genomic analysis of the importance of MAPK signaling in relation to the secretion apparatus during the defense process defense in the *G*. *max*-*H*. *glycines* pathosystem and identify additional targets for future studies.

## Introduction

Plants, like many organisms, respond to a number of biotic and abiotic challenges. These responses occur through complex signal transduction processes. Plants have a number of different signaling mechanisms that they use to respond to these challenges and included among them is the mitogen activated protein kinase (MAPK) cascade [1]. The MAPK cascade functions in eukaryotes by regulating many different cellular processes [2–4]. The MAPK cascade is a three-tiered signal transduction platform that is shared between eukaryotes [1]. It exists for cells to transduce input signals into a meaningful output [1]. Input information is processed through a stepwise series of phosphorylation events whereby MAP kinase kinase kinases (MAPKKKs) phosphorylate MAP kinase kinases (MAPKKs) that in turn phosphorylate MAPKs [5]. These events lead to an appropriate output response [5]. Due to this stepwise, shared processing, it has been determined that the MAPK cascade works as a cooperative enzyme, switching cells from one discrete state to another [6].

Many studies have shown the importance of the MAPK gene family functioning in plants during various processes including drought and cold stress as well as defense to pathogens [4, 7]. The ubiquitous nature of MAPKs and their roles in signaling also means they function in important ways in agricultural plants and among the most important is *Glycine max* (soybean). However, *G*. *max* has many pathogens that affect agronomic production and among the most important is the plant parasitic nematode *Heterodera glycines*, (soybean cyst nematode [SCN]). *H*. *glycines* causes more agronomic loss than the rest of its pathogens combined losses, creating an urgent need to understand and mitigate its pathogenicity [8].

With regard to pathogen defense, the MAPK3/6 genes have been the most extensively studied in plants [9, 10]. However, recent findings have expanded the breadth of MAPKs functioning in defense [11]. In those functional experiments performed in *G*. *max*, the entire 32 member MAPK gene family has been studied showing the *G*. *max* MAPK2, MAPK3-1, MAPK3-2, MAPK4-1, MAPK5-3, MAPK6-2, MAPK13-1, MAPK16-4 and MAPK20-2 have a defense role [11]. In a number of these cases, the MAPKs have been determined to be expressed within the *H*. *glycines*-induced feeding structure (syncytium) produced from a root pericycle cell that merges with 200–250 neighboring cells through the activities of the parasite. The syncytium is also the site of the localized defense response [11, 12]. Syncytium expression has been observed for MAPK2, MAPK3-1, MAPK4-1, MAPK5-3 and MAPK6-2, MAPK16-4 and MAPK20-2 [11]. Additional analyses of the expression of already proven defense genes, using quantitative real-time PCR (qRT-PCR), determined that a number of the defense MAPKs regulate the relative transcript abundance of already proven defense genes. The results are consistent with prior results that have examined the relative transcript abundances of various defense genes in this pathosystem [13, 14].

Within the *G*. *max*-*H*. *glycines* pathosystem, experiments have also demonstrated that the MAPKs function in ways that link or even converge in on pathogen associated molecular pattern (PAMP) triggered immunity (PTI) and effector-triggered immunity (ETI) branches of the defense response [11, 15]. Furthermore, experiments have demonstrated that the bacterial effector harpin is capable of inducing the expression of some of these defense MAPKs, including MAPK3-1, MAPK3-2 and MAPK5-3 [11, 16]. Experiments that have been presented in other plant pathosystems have shown that harpin is capable of engaging the transcription of the coiled-coil nucleotide binding leucine rich repeat (CC-NB-LRR) NON-RACE SPECIFIC DISEASE RESISTANCE 1 (NDR1)/HARPIN INDUCED1 (HIN1) which can transduce input signals through the MAPK pathway [16–19]. The same observations have been demonstrated in the *G*. *max*-*H*. *glycines* pathosystem [11, 20].

Also performing a role within this genetic defense network that is present in the *G*. *max*-*H*. *glycines* pathosystem is a homolog of *BOTRYTIS INDUCED KINASE1* (*BIK1*) [11, 13, 21]. The *A*. *thaliana* BIK1 functions as a receptor-like cytoplasmic kinase (RLCK) at a key branch point occurring between brassinosteroid ligand-facilitated growth signaling and defense signaling. This signaling is mediated by BRASSINOSTEROID INSENSITIVE 1 (BRI1) [22]. The defense branch of this signaling platform is mediated by several different microbe associated molecular pattern (MAMP) receptors. These receptors include FLAGELLIN SENSING 2 (FLS2) and EF-Tu RECEPTOR (EFR) as well as the *Arabidopsis* DAMP PEPTIDE 1 RECEPTOR (AtPEPR1). Each of these receptors are shared between BIK1 and BRI1-associated kinase 1 (BAK1) [21–25]. The experiments demonstrate the importance of BIK1 in mediating signals from a number of different effectors. Furthermore, the results are consistent with experiments that demonstrated *G*. *max* BIK1 is expressed specifically during a defense response within syncytia undergoing a resistant reaction and functions in defense [13, 26]. Recent results have also shown that the overexpression of the *G*. *max* BIK1 leads to induced expression of MAPK3-1 and MAPK3-2 [11].

The goal of the analysis presented here is to identify genes whose expression is regulated by MAPKs in *G*. *max* that function in defense to *H*. *glycines*, possibly aiding in understanding the convergence of ETI and PTI processes [27]. Toward this goal, previously generated RNA seq data has been analyzed from the transgenic defense MAPK-overexpressing (MAPK-OE) lines generated in *G*. *max* [28]. The analysis has resulted in the identification of a core set of 309 genes that are induced in common between the different MAPK-OE lines (referred to as MAPK-OE-all). Those induced genes then have been compared to previously generated data that identified genes that have been determined to exhibit expression within *H*. *glycines*-induced syncytia undergoing a defense response in two different *G*. *max* genotypes that are capable of undergoing two different forms of a defense reaction [11, 29]. The 71 identified genes have been further analyzed for the presence of a secretion signal since earlier studies have demonstrated the importance of the *G*. *max* secretion system to defense in this pathosystem [13, 14, 26, 30]. The analysis has resulted in the identification of 8 candidate defense genes that are induced in the MAPK-OE-all lines, are expressed within the syncytial cells undergoing the process of defense and are predicted to have secretion signals. These 8 candidate defense genes, identified from this pool, have been functionally tested through transgenic experiments and show they have a defense role. We note here that these are not the only genes that appear to have a role in the defense process, but have been examined as part of a larger ongoing series of studies to better understand the defense process in the *G*. *max*-*H*. *glycines* pathosystem.

## Materials and methods

### Genetic stocks

The MAPKs that have a defense function against *H*. *glycines* have been described [11]. Overexpression of the *G*. *max* defense MAPKs (MAPKs) has been performed in the *H*. *glycines* susceptible line *G*. *max*_[Williams 82/PI 518671]_ [11]. The growth, culture of the genetic lines, RNA isolations and confirmation of the expression of the genetic constructs have been described in McNeece et al. [11].

### RNA seq analyses

The RNA sequencing (RNA seq) data used in this analysis has been obtained from our prior study and is available as BioProject ID PRJNA664992, Submission ID: SUB8182387 [28]. Single replicate generation of RNA seq data of RNA isolated from the 9 MAPK-OE lines, including MAPK2 (Glyma.06G029700), MAPK3-1 (Glyma.U021800), MAPK 3–2 (Glyma.12G073000), MAPK 4–1 (Glyma.07G066800), MAPK 5–3 (Glyma.08G017400), MAPK6-2 (Glyma.07G206200), MAPK 13–1 (Glyma.12G073700), MAPK16-4 (Glyma.07G255400) and MAPK20-2 (Glyma.14G028100) and controls has been performed by Omega Bioservices, 400 Pinnacle Way, Ste 425, Norcross, GA 30071 and described [11, 28]. RNA sequences have been deposited and made publicly available in the MAPKDB: A MAP kinase database for signal transduction element identification) [28]. The MAPKDB database, storing essential RNA seq data relating to the *G*. *max* MAPK-OE and RNAi experiments, allows data to be retrievable based on gene identification (gene ID). The MAPK database stores descriptions of every gene that has been obtained from the RNA seq study samples, including eukaryotic orthologous groups (KOG), gene ontology (GO) assignments and protein families (PFAM). A Microsoft SQL Server 2016 Enterprise Edition has been used to design, implement and host the developed MAPKDB. The MAPKDB, as it is designed, allows users to browse, search and download the data using gene IDs or descriptions. The sample differential gene expression results are compared by the users [11].

### Detection call methodology (DCM)

The relevant p-values obtained from the DCM analysis examining RNA obtained from laser-microdissected (LM) root cells performed in Matsye et al. [26] are provided here as supplemental data. The DCM procedure has been presented, but some details are provided for analysis methodology clarity [26, 29]. Two different *H*. *glycines*-resistant genotypes *G*. *max*_[Peking/PI 548402]_ and *G*. *max*_[PI 88788]_ have been infected with *H*. *glycines*_[NL1-Rhg/HG-type 7/race 3]_ [26, 29]. The infections have led to the expected resistant outcome as proven through histocytological analyses [26, 29]. LM has been used to collect pericycle cells at a time point prior to *H*. *glycines* infection (the 0 days post infection [dpi] control) [26, 29]. In those same analyses, syncytia undergoing the process of resistance at 3 and 6 dpi have been collected by LM [26, 29]. The cDNA probes have been produced from the 0, 3 and 6 dpi RNA samples to be used in hybridization experiments to the Affymetrix® Soybean Gene Chip® [26, 29]. The experiments using the probe that has been generated for each soybean genotype (*G*. *max*_[Peking/PI 548402]_ and *G*. *max*_[PI 88788]_) and have been run in three independent biological replicates. This process has resulted in the production of 6 total arrays for each time point (3 arrays for *G*. *max*_[Peking/PI 548402]_ and 3 arrays for *G*. *max*_[PI 88788]_). The DCM microarray-generated gene expression data that has been obtained from the syncytium samples undergoing two different forms of a defense response have originally been analyzed using Bioconductor®. The microarray expression levels that have been measured by the probe sets have been extracted using the Robust Multichip Average (RMA) methodology as implemented in the Affymetrix® Bioconductor® package consisting of (1) convolution background correction, (2) quantile normalization, and (3) summarization based upon a multi-array model of the normalized and log (base 2) transformed probe set data. The presence or absence of a particular probe set’s (gene’s) transcript on a single array have been determined using the Bioconductor® implementation of the standard Affymetrix® DCM. DCM consists of four steps. The DCM consists of (1) removal of saturated probes, (2) calculation of discrimination scores, (3) p-value calculation using the Wilcoxon’s rank test, and (4) making the detection call. The algorithm determines if the presence of a probe set’s transcript is provably different from zero (present [P]), uncertain (marginal [M]), or not provably different from zero (absent [A]). Measurable (M) expression is a p value less than 0.05 (p < 0.05). Not measurable expression (NM) is a p value greater than or equal to 0.05 (p ≥ 0.05). The gene expression data for the MAPK-OE-all induced gene expression data has been extracted from this data set. An important aspect of these studies is that due to how the Affymetrix ® microarray has been constructed, some genes have had no probe set fabricated onto the array. Consequently, gene expression could not be quantified for those genes according to the analysis procedures. In these cases, the gene expression analysis is not applicable (n/a) to those genes. Genes lacking Affymetrix® probe sets have not been considered further in the analysis presented here.

### Gene Ontology analysis

Gene Ontology (GO) analyses have been performed on the protein sequences composing the list of 309 induced genes. The GO analyses have been retrieved from Phytozome. The specific tool used was PhytoMine (https://phytozome.jgi.doe.gov/phytomine/begin.do). Graphs have been generated using Excel. The analyses of the MAPK-OE-all induced and suppressed genes have been divided into three different categories including biological process, molecular function and cellular component. The same analyses have been performed for the 71 genes that are both induced in the MAPK-all-OE transgenic lines and expressed within the syncytium in the (*G*. *max*_[Peking/PI 548402]_ and *G*. *max*_[PI 88788]_) genotypes.

### The cloning of candidate defense genes into the destination vectors

The pRAP destination vectors are based off of the published Gateway® cloning vector platform [11, 30, 31]. The candidate defense gene amplicons that have been generated by polymerase chain reaction (PCR) have been ligated in the pENTR/D-TOPO® entry vector (Invitrogen®) according to the manufacturer’s instructions. The PCR primer sequences that have been used in the reactions are provided (**S1 Table**). LR Clonase® (Invitrogen®) has been used to facilitate the transfer the candidate resistance gene amplicon to the pRAP15 overexpression and pRAP17 RNAi destination vectors according to the manufacturer’s instructions. As the controls for the experiments, the un-engineered pRAP15 or pRAP17 vectors have the *ccd*B gene located in the position where, otherwise, the candidate resistance gene amplicon would be inserted during the LR clonase reaction. As a result, this feature makes the pRAP15-*ccd*B (overexpression control) and pRAP17-*ccd*B (RNAi control) un-engineered vectors suitable controls for any non-specific effects caused by gene overexpression or RNAi [11]. The destination vectors have been used in freeze-thaw incubation experiments to transform chemically competent *Agrobacterium rhizogenes* K599 (K599) with the designated gene cassette [11].

### Production of transgenic plants

The vectors and procedures have been published [30, 31]. Visual selection of transgenic *G*. *max* roots is performed with the enhanced green fluorescent reporter (eGFP) [11]. Roots exhibiting the eGFP reporter expression will also possess the candidate defense gene expression cassette, each with their own promoter and terminator sequences. K599 transfers the DNA cassettes located between the left and right borders of the pRAP15 and pRAP17 destination vectors into the root cell chromosomal DNA. The result is a stable transformation event in the root somatic cells even though the construct is not incorporated into the germline. Roots subsequently develop from this transgenic cell over a period of a few weeks from the base of the shoot stock. The resultant genetic mosaic has a non-transgenic shoot having a transgenic root system so in the experiments presented here, each individual transgenic root system is an independent transformant line. The transgenic plants are planted in SC10 Super cone-tainers (Stuewe and Sons, Inc.®) that are secured in RL98 trays (Stuewe and Sons, Inc.®) which are allowed to recover for two weeks prior to the start of the experiment [11]. The functionality of the genetic constructs in *G*. *max* is confirmed by qRT-PCR (Please refer to quantitative PCR [qRT-PCR] section).

### The synthesis of cDNA

The method has been performed according to McNeece et al. [11]. *G*. *max* root mRNA has been isolated using the UltraClean® Plant RNA Isolation Kit (Mo Bio Laboratories®, Inc.) according to the manufacturer’s instructions [11]. The removal of genomic DNA from the mRNA is done with DNase I (Invitrogen®) according to the manufacturer’s instructions. SuperScript® First Strand Synthesis System for RT-PCR (Invitrogen®) using the oligo d(T) as the primer is used to synthesize the cDNA from mRNA according to the manufacturer’s instructions.

### Quantitative real-time PCR (qRT-PCR) assessment of gene expression

The method has been performed according to McNeece et al. [11]. Assessment of candidate resistance gene expression in *G*. *max* has been accomplished by qPCR. The methods have used Taqman® 6-carboxyfluorescein (6-FAM) probes and Black Hole Quencher (BHQ1) (MWG Operon; Birmingham, AL) (**S1 Table**) [11]. The qPCR control, used in the *G*. *max* experiments, has been designed from a ribosomal S21 protein coding gene (Glyma.15G147700) [11]. The fold change in gene expression that has been caused by the genetic engineering event has been calculated using 2^-ΔΔ*C*^_T_ [11, 32]. A Student’s *t*-test has been used to calculate the p-values for the replicated qPCR reactions [33]. The procedures follow those presented by McNeece et al. [11].

### Assaying the effect the genetic engineering event has on nematode parasitism

The infections of the transgenic plants by *H*. *glycines* has been performed according to the procedures described in McNeece et al. [11]. The female index (FI) has been calculated by procedures originally described by Golden et al. [34] and employed for transgenic research [11]. The FI is the community-accepted standard representation of the obtained data [11]. The FI = (Nx/Ns) X 100. In the procedure employed here, Nx is the pRAP-gene-transformed (experimental [candidate resistance gene]) line. Ns is the pRAP15/17-*ccd*B (control) line [11]. The FI has been calculated as cysts per mass of the whole root (wr) and also cysts per gram (pg) of root [11]. The whole root analysis is how the data has been presented historically [34]. The cyst per gram analysis accounts for possible altered root growth caused by the influence of the overexpression or RNAi of the candidate defense gene. Three independent biological replicates have been made for each construct with 10–20 individual transgenic plants each serving as experimental replicates within each biological replicate, statistically examined using the Mann–Whitney–Wilcoxon (MWW) Rank-Sum Test, p < 0.05 cutoff [11, 35]. The MWW Rank-Sum Test is a nonparametric test of the null hypothesis not requiring the assumption of normal distributions [35].

### Bioinformatics

Signal peptide prediction has been performed for the conceptually translated selected candidate defense genes. The procedure has employed SignalP-5.0 [36]. The procedure has used the default settings to identify the likelihood (p ≥ 0.5) of having a predicted signal peptide. There are three types of peptides that can be identified, including a Sec signal peptide (Sec/SPI), a Lipoprotein signal peptide (Sec/SPII), a Tat signal peptide (Tat/SPI). Furthermore, No signal peptide at all (Other) could be determined [36].

### Analysis of the transgenic constructs on root mass

Independent root growth analyses have been done in three biological replicates on one month old roots excised from the stem with fresh weight measurements made according to [11]. The percent difference in root weight has been calculated by taking the average weight of 10 candidate gene OE and 10 candidate gene RNAi cassette-expressing root masses for the target as compared to the respective 10 pRAP15-OE and pRAP17-RNAi controls, multiplied by 100. The p-values have been calculated by a Student’s t-test [33].

## Results

### Defense MAPK gene expression

**Fig 1:**
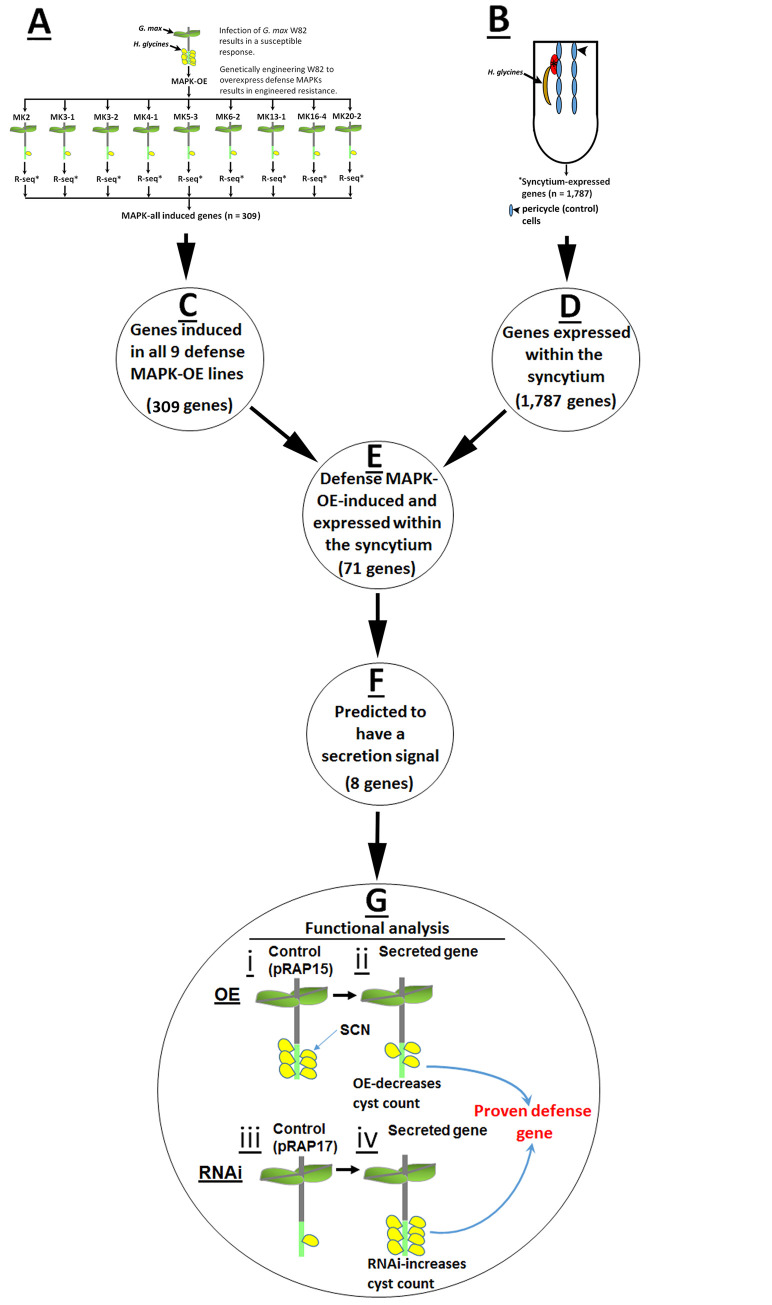
Analysis pipeline. **A.** The defense MAPKs have been identified through transgenic analyses of all 32 members of the gene family [11]. RNA has then been isolated from the MAPK-OE defense-MAPK lines as well as their accompanying pRAP15 controls for RNA seq analyses [28]. **B.** In an associated analysis of the gene expression occurring within the syncytium (upper right panel), two different *G*. *max* genotypes that are capable of a defense response (*G*. *max*_[Peking/PI 548402]_ and *G*. *max*_[PI 88788]_) have been analyzed. **C.** The RNA seq study has identified 309 genes that are induced in common between each of the MAPK-OE lines (MAPK-OE-all). **D.** The 1,787 syncytium-expressed gene list has been to be used to identify whether any of those genes are regulated by MAPKs (shown in C). **E.** A comparison of C and D gene lists has resulted in the identification of 71 genes that are in common between the MAPK-all and syncytium expressed gene analyses. **F.** Prior analyses have demonstrated the *G*. *max* secretion apparatus is important in defense to *H*. *glycines* parasitism [13, 14, 30]. The 71 candidate defense genes have been further narrowed down for the functional studies by conceptually translating their protein products to determine if any are predicted to be secreted proteins. The analyses has employed SignalP-5.0 leading to the identification of 8 (of the 71) genes that have a predicted secretion signal [36]. These 8 genes became the candidate defense genes that have been tested in transgenic studies presented here. Please see the Materials and Methods section for analysis details.

An analysis pipeline has been developed to identify genes that (1) are induced in common between each of the MAPK-OE lines and (2) are expressed in *H*. *glycines* syncytia undergoing the process of defense and (3) have secretion signals (**Fig 1**). RNA seq data has been generated from RNA isolated from the *G*. *max* defense MAPK-OE lines for MAPK2, MAPK3-1, MAPK3-2, MAPK4-1, MAPK5-3, MAPK6-2, MAPK13-1, MAPK16-4 and MAPK20-2 [28]. In analyses presented here, genes having induced or suppressed relative transcript abundances as compared to the pRAP15 engineered control have been determined for each MAPK-OE line (**Table 1, S2 Table**). The study has determined that there is a core set of genes whose expression is influenced by the overexpression of each of the defense MAPKs (MAPK-OE-all). The analysis has identified 309 genes with higher relative levels of transcript abundance as compared to the pRAP15-expressing control (p < 0.05) (**Table 1, S3 Table**). In contrast, 815 genes have been identified to have lower relative levels of transcript abundance as compared to the pRAP15-expressing control (p < 0.05) (**S4 Table**).

**Table 1:** Detection call methodology as compared to the MAPK-OE-all induced genes, leading to the identification of 71 genes having measured expression in each analysis.

Name	MK-(I)*	SP**	Gene Description	Time point***			Expression type
				0	3	6	
Glyma.19G020700	Y	**Y**	Galactose mutarotase-like superfamily protein	NM	M	M	**Type 1 (n = 16)**
Glyma.13G178700	Y	**Y**	Pollen Ole e 1 allergen and extensin family protein	NM	M	M	
Glyma.09G096700	Y	**Y**	Endomembrane protein 70 protein family	NM	M	M	
Glyma.14G020000	Y	**Y**	O-Glycosyl hydrolases family 17 protein	NM	M	M	
Glyma.13G349300	Y	**Y**	Glycosyl hydrolases family 32 protein	NM	M	M	
Glyma.01G231900	Y	N	Rieske (2Fe-2S) domain-containing protein	NM	M	M	
Glyma.06G025200	Y	N	lysine decarboxylase family protein	NM	M	M	
Glyma.09G085600	Y	N	AWPM-19-like family protein	NM	M	M	
Glyma.05G148300	Y	N	Calmodulin-binding transcription activator protein	NM	M	M	
Glyma.05G138800	Y	N	Cytochrome b561/ferric reductase transmembrane protein family	NM	M	M	
Glyma.18G018600	Y	N	myo-inositol-1-phosphate synthase 2	NM	M	M	
Glyma.19G091400	Y	N	Ferritin/ribonucleotide reductase-like family protein	NM	M	M	
Glyma.02G301800	Y	N	oligopeptide transporter 5	NM	M	M	
Glyma.03G114700	Y	N	Plant protein of unknown function (DUF247)	NM	M	M	
Glyma.08G365900	Y	N	Mannose-6-phosphate isomerase, type I	NM	M	M	
Glyma.14G104100	Y	N	monogalactosyl diacylglycerol synthase 1	NM	M	M	
Glyma.12G096300	Y	**Y**	FASCICLIN-like arabinogalactan protein 17 precursor	NM	NM	M	**Type 2 (n = 25)**
Glyma.01G171100	Y	**Y**	Peroxidase superfamily protein	NM	NM	M	
Glyma.12G064300	Y	**Y**	Pathogenesis-related thaumatin superfamily protein	NM	NM	M	
Glyma.07G054800	Y	N	auxin response factor 18	NM	NM	M	
Glyma.18G117100	Y	N	Basic-leucine zipper (bZIP) transcription factor HY5	NM	NM	M	
Glyma.08G302500	Y	N	Basic-leucine zipper (bZIP) transcription factor HY5	NM	NM	M	
Glyma.01G022500	Y	N	AINTEGUMENTA-like 6	NM	NM	M	
Glyma.10G037500	Y	N	ARM repeat superfamily protein	NM	NM	M	
Glyma.11G230100	Y	N	phospholipase C 2	NM	NM	M	
Glyma.17G144700	Y	N	homeobox protein 2	NM	NM	M	
Glyma.13G220500	Y	N	BCL-2-associated athanogene 2	NM	NM	M	
Glyma.03G122000	Y	N	cytochrome P450, family 98, subfamily A, polypeptide 3	NM	NM	M	
Glyma.14G221200	Y	N	cinnamyl alcohol dehydrogenase 9	NM	NM	M	
Glyma.04G038800	Y	N	Vacuolar import/degradation, Vid27-related protein	NM	NM	M	
Glyma.05G179800	Y	N	Haloacid dehalogenase-like hydrolase (HAD) superfamily protein	NM	NM	M	
Glyma.02G310600	Y	N	Protein of unknown function (DUF630 and DUF632)	NM	NM	M	
Glyma.17G062600	Y	N	AINTEGUMENTA-like 5	NM	NM	M	
Glyma.15G066500	Y	N	Major facilitator superfamily protein	NM	NM	M	
Glyma.11G034000	Y	N	tonoplast intrinsic protein 2;3	NM	NM	M	
Glyma.06G116400	Y	N	amino acid permease 3	NM	NM	M	
Glyma.12G192600	Y	N	B-cell receptor-associated 31-like	NM	NM	M	
Glyma.13G107900	Y	N	nuclear factor Y, subunit A3	NM	NM	M	
Glyma.06G029100	Y	N	BEL1-like homeodomain 3	NM	NM	M	
Glyma.19G114700	Y	N	dehydrin	NM	NM	M	
Glyma.13G049700	Y	N	Methylenetetrahydrofolate reductase family protein	NM	NM	M	
Glyma.12G150500	Y	N	Aluminium induced protein with YGL and LRDR motifs	NM	M	NM	**Type 3 (n = 4)**
Glyma.02G029900	Y	N	UDP-glucosyl transferase 74B1	NM	M	NM	
Glyma.19G214600	Y	N	C2H2 and C2HC zinc fingers superfamily protein	NM	M	NM	
Glyma.14G039800	Y	N	no description	NM	M	NM	
Glyma.20G133100	Y	N	Phototropic-responsive NPH3 family protein	M	NM	NM	**Type 4 (n = 5)**
Glyma.01G211000	Y	N	Glycosyl hydrolases family 32 protein	M	NM	NM	
Glyma.20G181900	Y	N	Transmembrane amino acid transporter family protein	M	NM	NM	
Glyma.09G179900	Y	N	Heavy metal transport/detoxification superfamily protein	M	NM	NM	
Glyma.17G257200	Y	N	Putative lysine decarboxylase family protein	M	NM	NM	
Glyma.04G233400	Y	N	Transducin/WD40 repeat-like superfamily protein	M	NM	M	**Type 5 (n = 5)**
Glyma.15G044400	Y	N	related to AP2.7	M	NM	M	
Glyma.13G141900	Y	N	myb domain protein 36	M	NM	M	
Glyma.06G035300	Y	N	cytochrome P450, family 82, subfamily C, polypeptide 4	M	NM	M	
Glyma.05G146300	Y	N	ARM repeat superfamily protein	M	NM	M	
Glyma.16G037900	Y	N	no description	M	M	M	**Type 6 (n = 16)**
Glyma.15G007500	Y	N	Protein of unknown function (DUF567)	M	M	M	
Glyma.09G254800	Y	N	WRKY family transcription factor	M	M	M	
Glyma.10G249100	Y	N	PLAT/LH2 domain-containing lipoxygenase family protein	M	M	M	
Glyma.02G260800	Y	N	EARLY RESPONSIVE TO DEHYDRATION-15	M	M	M	
Glyma.09G118800	Y	N	plastid movement impaired protein	M	M	M	
Glyma.19G198900	Y	N	NDR1/HIN1-like 1	M	M	M	
Glyma.08G336800	Y	N	amino acid permease 7	M	M	M	
Glyma.06G249800	Y	N	PYR1-like 12	M	M	M	
Glyma.02G080900	Y	N	cellulose synthase 6	M	M	M	
Glyma.03G158100	Y	N	histone H2A 11	M	M	M	
Glyma.15G017000	Y	N	Major facilitator superfamily protein	M	M	M	
Glyma.04G243000	Y	N	Thiamin diphosphate-binding fold (THDP-binding) superfamily protein	M	M	M	
Glyma.13G296900	Y	N	two-pore channel 1	M	M	M	
Glyma.19G091100	Y	N	DCD (Development and Cell Death) domain protein	M	M	M	
Glyma.11G097500	Y	N	Calcium-binding EF-hand family protein	M	M	M	

**Footnote**: MK-(I)*, MAPK-all induced expression; Y, yes.

**Footnote**: SP**, Y (yes), has a signal peptide; N (no) does not have a signal peptide as described in the Materials and Methods section.

**Footnote**: Only genes with expression in at least one of the three selected time points*** (0, 3 or 6 dpi).

**Footnote**: The genes highlighted in orange are those having predicted secretion signals.

**Footnote**: Time points highlighted in blue do not meet the criteria set for measured (M) expression and are considered not measured (NM).

**Footnote**: Time points highlighted in red do meet the criteria set for measured (M) expression and are considered measured (M).

**Footnote**: Expression type (Types 1–6) is based off of the profile determined from the expression that has been measured at the 0, 3 and 6 dpi time points.

### Gene Ontology analysis for the MAPK-all induced accessions

**Fig 2:**
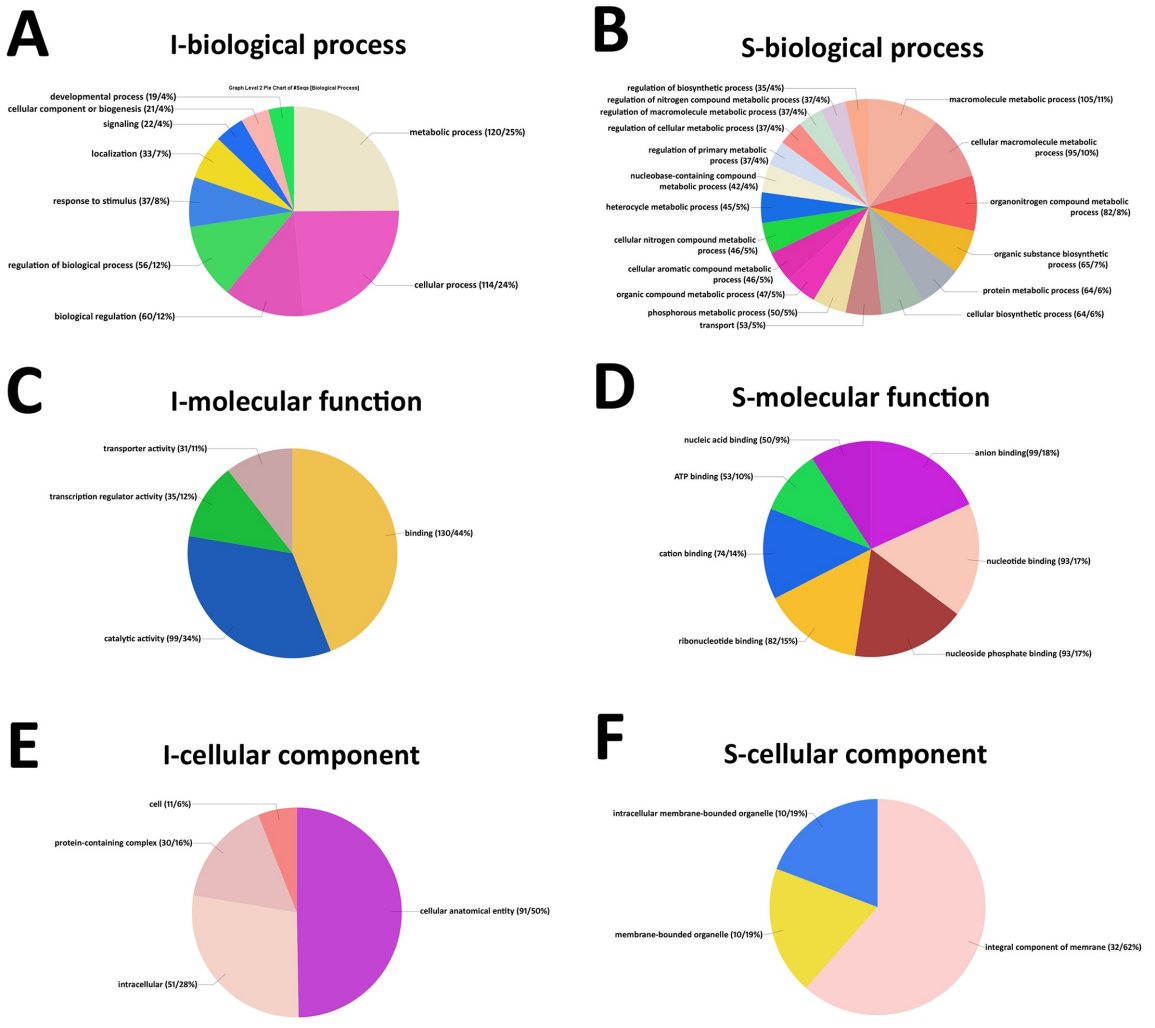
The Gene Ontology analysis of the 309 MAPK-OE-all genes. **A.** Induced genes GO analysis-biological process. **B.** Suppressed genes GO analysis-biological process. **C.** Induced genes GO analysis-molecular function. **D.** Suppressed genes GO analysis-molecular function. **E.** Induced genes GO analysis-cellular component. **F.** Suppressed genes GO analysis-cellular component. Gene Ontologies, specifically molecular function, have been retrieved from Phytozome, using the PhytoMine tool (https://phytozome.jgi.doe.gov/phytomine/begin.do). Graphs have been generated using Excel.

To obtain a clearer understanding of the biological role of the identified genes, Gene Ontology (GO) analyses have been performed on these conceptually translated genes composing the list of 309 MAPK-OE-all-induced and 815 MAPK-OE-all-suppressed genes. Graphs that represent the biological process, molecular function and cellular component for the genes are provided (**Fig 2**). The 309 MAPK-OE-all-induced genes became the focus of the remaining analyses.

### Comparative analyses of MAPK-OE-all induced accessions to syncytium-expressed accessions

Comparative analyses have been performed that focus in on the 309 genes comprising the MAPK-OE-all induced RNA seq gene expression data in comparison to the 1,787 syncytium expressed genes [26, 29]. The comparisons have resulted in the identification of a set of 167 genes (54.0%) that have Affymetrix® microarray probe set identifiers assigned to them (**Table 1, S5 Table**). These genes have been divided into 7 types (Types 1–7), based on the expression profile that has been generated by the data obtained from the laser microdissection experiment of the 0, 3 and 6 dpi time point syncytium RNA samples. Of the 167 genes that have expression in the MAPK-OE-all and also have Affymetrix® probe set identifiers, 71 genes (42.51%) are observed to be expressed in at least one of the three selected time points. These three time points are 0 (control), 3 or 6 days post *H*. *glycines* infection (dpi) that relate to the syncytium gene expression study (**Table 1**). Of these 71 genes, 45 (63.38%) do not exhibit expression prior to infection and cluster as Types 1–3 (**Table 1**). The Type 1 category is defined by gene expression having not been measured at the 0 dpi time point (p ≥ 0.05), but has been measured at the 3 and 6 dpi time points (p < 0.05). There are 16 genes (22.54%) that have been classified as having a Type 1 expression profile (**Table 1**). The Type 2 category is characterized by gene expression not being measured at the 0 dpi time point (p ≥ 0.05), but has been measured at the 6 dpi time point (p < 0.05) (**Table 1**). There are 25 genes (35.21%) exhibiting Type 2 gene expression (**Table 1**). The Type 3 category is characterized by gene expression not being measured at the 0 or 6 dpi time points (p ≥ 0.05), but has been measured at the 3 dpi time point (p < 0.05) (**Table 1**). Type 3 expression has been observed for 4 genes (5.63%) (**Table 1**). The remaining 26 genes exhibit expression patterns that do include expression at the 0 dpi time point and span Types 4–6 (p < 0.05) (**Table 1**). The Type 4 category is characterized by genes expressed at the 0 dpi time point only (p < 0.05) (**Table 1**). There are 5 genes (7.04%) exhibiting Type 4 expression (**Table 1**). The Type 5 category is characterized by gene expression at the 0 and 6 dpi time points (p < 0.05), but not at the 3 dpi time point (p ≥ 0.05) (**Table 1**). There are 5 genes (7.04%) exhibiting Type 5 expression (**Table 1**). The Type 6 category is characterized by expression occurring at the 0, 3 and 6 dpi time points (p < 0.05) (**Table 1**). There are 16 genes (22.54%) exhibiting Type 6 expression (**Table 1**). The remaining 96 genes, of the 167 genes having Affymetrix® probe sets (57.49%) that make up the Type 7 category, having not been observed to be expressed in the 0, 3 or 6 dpi time points (p ≥ 0.05) (**Table 1, S6 Table**).

### Gene Ontology (GO) analyses

**Fig 3:**
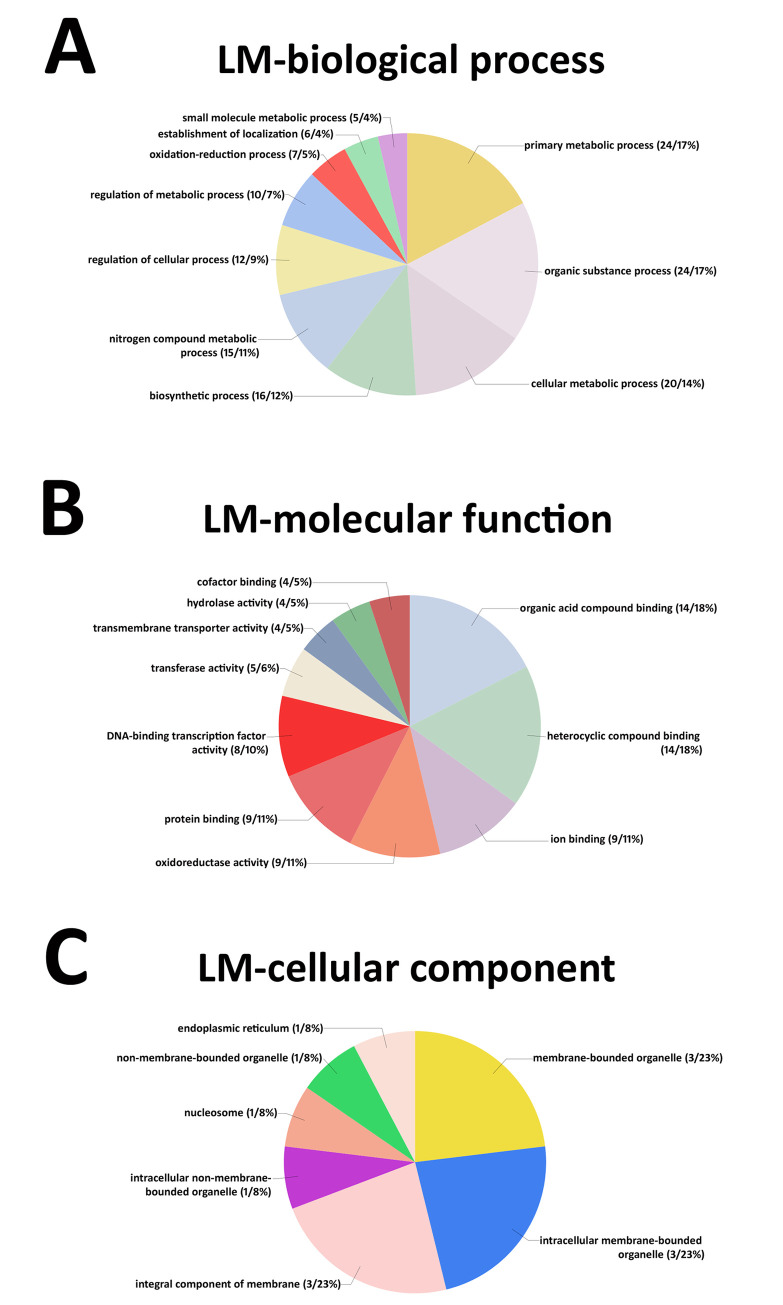
The Gene Ontology analysis of the 71 MAPK-OE-all genes identified from the laser microdissected (LM) syncytium cell analyses and the MAPK-OE-all genes induced gene analyses. The analyses included the 0 dpi (control), 3 and 6 dpi LM syncytia undergoing a defense response in *G*. *max*_[Peking/PI 548402]_ and *G*. *max*_[PI 88788]_. **A.** GO analysis-biological process. **B.** GO analysis-molecular function. **C.** GO analysis-cellular component. Gene Ontologies, specifically molecular function, have been retrieved from Phytozome, using the PhytoMine tool (https://phytozome.jgi.doe.gov/phytomine/begin.do). Graphs have been generated using Excel.

GO analyses have been performed on the 71 MAPK-all-OE induced genes that also have Affymetrix® probe set identifiers and are observed to be expressed in at least one of the three selected time points. The analyses have been divided into separate graphs that represent the biological process, molecular function and cellular component. The analyses are presented (**Fig 3**).

### Signal peptide prediction of the MAPK-OE-all expressed genes

Analyses have demonstrated the *G*. *max* secretion system functions in its defense process toward *H*. *glycines* parasitism [13, 14, 26, 30]. To examine this concept further, the 167 genes that have been identified as being expressed in the MAPK-all-OE RNA seq studies have been examined subsequently to determine if they have characteristics of secreted proteins. Putatively secreted proteins have been identified through the prediction of a signal peptide using the signal peptide detection program SignalP-5.0 [36]. The analyses have resulted in the identification of 8 of the 71 syncytium-expressed genes (11.27%) whose conceptually translated cDNA sequences are likely to have secretion signals (**Table 1, Fig 1, S1 Fig**). The 8 genes can be divided into two groups. The first group has been found to be expressed at 3 and 6 dpi syncytium, but not in the 0 dpi control (Type 1). The 5 Type 1 genes that have been predicted to have signal peptides include galactose mutarotase-like superfamily protein (Glyma.19G020700), extensin (Glyma.13G178700), endomembrane protein 70 (Glyma.09G096700), O-glycosyl hydrolases family 17 (Glyma.14G020000) and glycosyl hydrolases family 32 (Glyma.13G349300). The second group has been found to be expressed in the 6 dpi syncytium, but not in the 3 dpi syncytium or 0 dpi control (Type 2). The 3 Type 2 proteins (4.23%) that have been predicted to have signal peptides include FASCICLIN-like arabinogalactan protein 17 precursor (Glyma.12G096300), secreted peroxidase superfamily protein (Glyma.01G171100) and pathogenesis-related thaumatin superfamily protein (Glyma.12G064300) (**Table 1**). No genes exhibiting characteristics of secreted proteins have been identified for expression Types 3–6.

The 97 proteins that have not been identified to be expressed within the 0 dpi control, 3 or 6 dpi syncytium (Type 7) have also been examined. The results of those analyses have led to the identification of 11 (11.34%) genes that have predicted signal peptides (**Table 1**). The accessions include Barwin-like endoglucanases superfamily protein (Glyma.07G132600), Eukaryotic aspartyl protease family protein (Glyma.17G016600), Peroxidase superfamily protein (Glyma.09G277800), Bifunctional inhibitor/lipid-transfer protein/seed storage 2S albumin superfamily protein (Glyma.11G120400), PEP1 receptor 1 (Glyma.10G195700), root hair specific 19 (Glyma.05G103600); GAST1 protein homolog 4 (Glyma.19G022500), glycosylphosphatidylinositol-anchored lipid protein transfer 1 (Glyma.11G251100), expansin 11 (Glyma.17G260400), D-arabinono-1,4-lactone oxidase family protein (Glyma.16G020100) and bifunctional inhibitor/lipid-transfer protein/seed storage 2S albumin superfamily protein (Glyma.18G191600) (**Table 1, S6 Table**). These accessions that have been determined to not be expressed within the syncytium are beyond the scope of the present study.

### Preparation of transgenic plants

**Fig 4:**
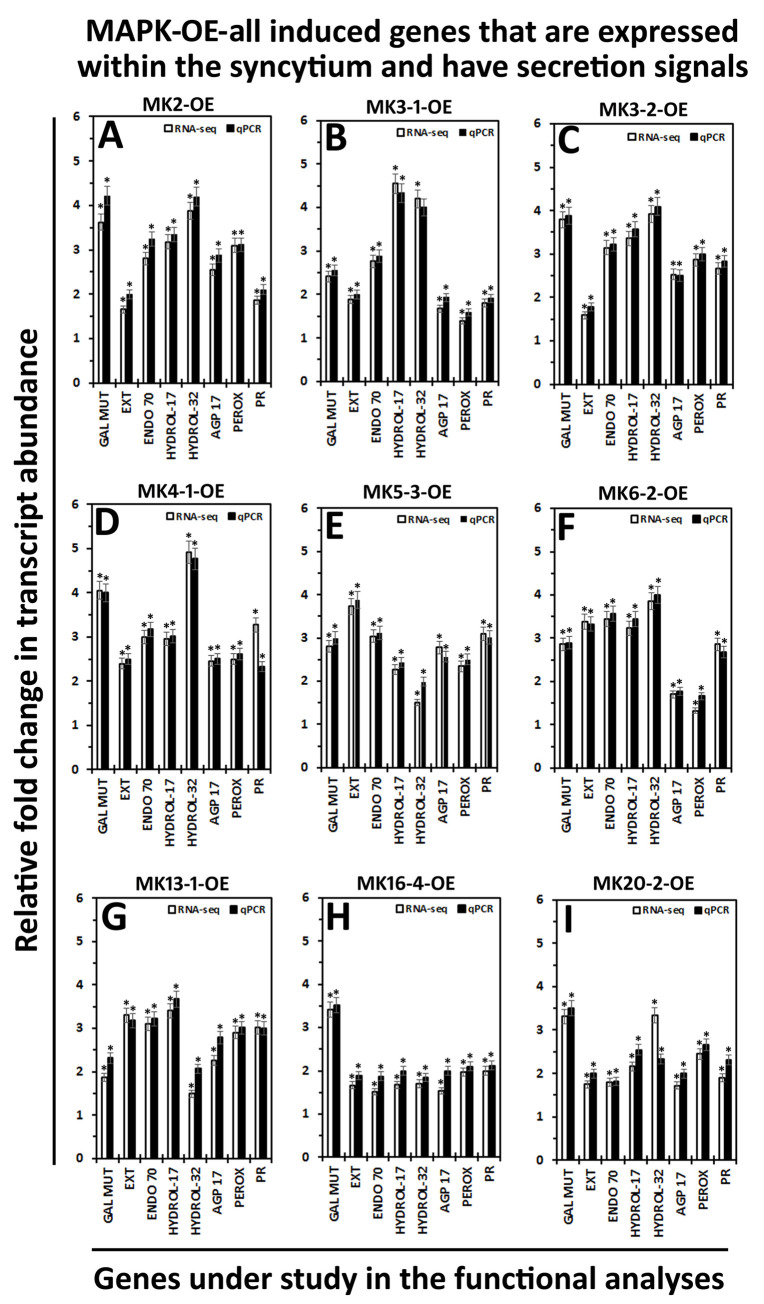
A qRT-PCR confirmation of the RNA seq gene expression data occurring for the 8 genes targeted for transgenic functional analyses that are MAPK-OE-all expressed, syncytium-expressed and are putatively secreted. The target genes are a galactose mutarotase-like (GAL MUT, Glyma.19G020700), Pollen Ole e 1 allergen and extensin family protein (EXT, Glyma.13G178700); endomembrane protein 70 protein family (ENDO 70, Glyma.09G096700); O-Glycosyl hydrolases family 17 protein (HYDROL-17, Glyma.14G020000); glycosyl hydrolases family 32 protein (HYDROL-32, Glyma.13G349300); FASCICLIN-like arabinogalactan protein 17 precursor (AGP 17, Glyma.12G096300); peroxidase superfamily protein (PEROX, Glyma.01G171100); pathogenesis-related thaumatin superfamily protein (PR, Glyma.12G064300). A minimum cutoff is set at ± 1.5 fold. * Statistically significant. The p-values (p < 0.001) for the replicated qPCR analyses have been calculated through a Student’s *t*-test [33]. Please refer to Methods for analysis details. The qPCR analyses have been averaged for 3 independent replicates. MK = MAPK.

The 5 Type 1 and 3 Type 2 putatively secreted proteins, selected from the MAPK-All-OE syncytium-expressed accessions having predicted secretion signals, became the candidates for the transgenic study. Prior to the transgenic experiments, the expression of the 8 candidate genes have been confirmed in the defense MAPK-OE transgenic lines that have been used originally to generate the RNA-seq data. The quantitative real time PCR (qRT-PCR)-based experiments confirm that the candidate defense genes are induced in their expression in each of the defense MAPK-OE transgenic lines (P < 0.05) (**Fig 4**).

**Fig 5:**
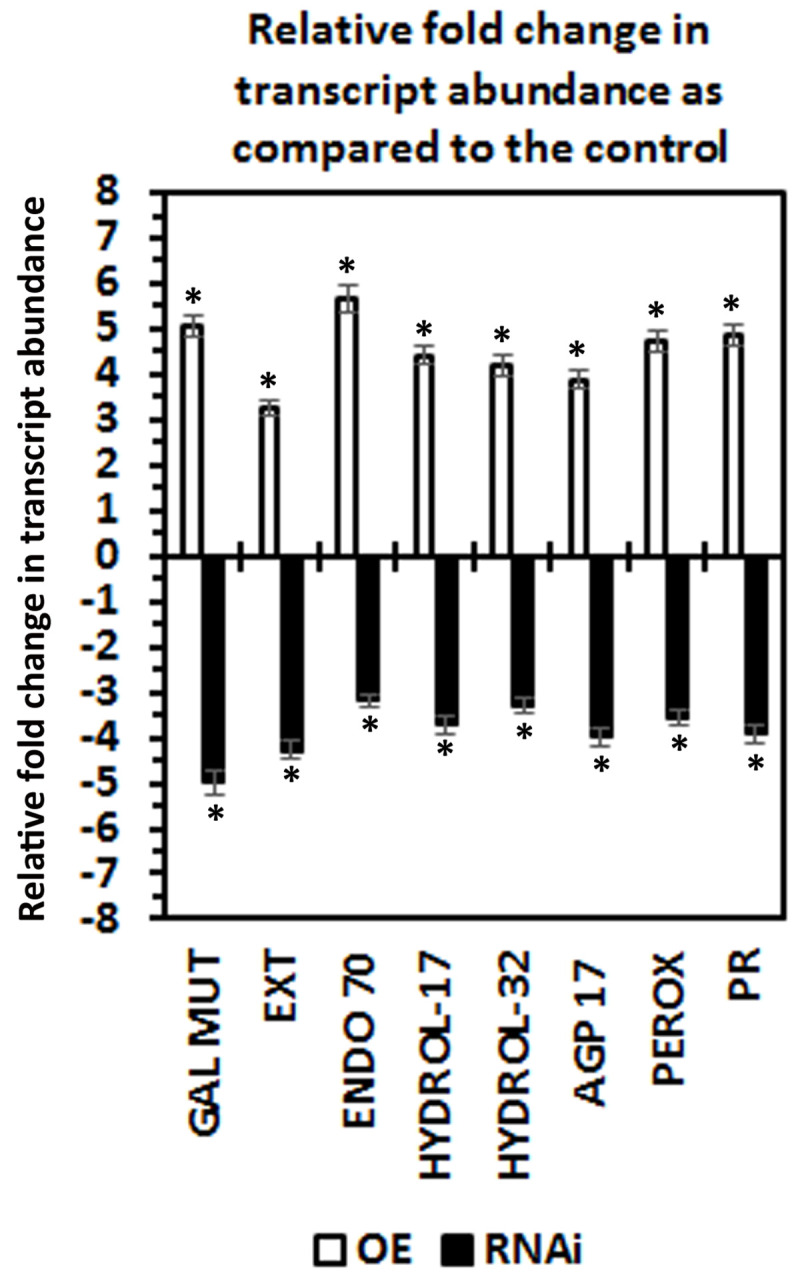
Confirmation of effect the candidate defense gene expression cassette (OE or RNAi) has on the relative level of expression of the target gene. The p-values (p < 0.001) for the replicated qPCR analyses have been calculated through a Student’s *t*-test [33]. Please refer to Methods for analysis details. The qPCR analyses have been averaged for 3 independent replicates.

The genes subsequently have been genetically engineered for their overexpression in the *H*. *glycines*-susceptible *G*. *max*_[Williams 82/PI 518671]_. These experiments have been performed to determine if an engineered defense response could be obtained. In contrast, the candidate genes have then been genetically engineered for their RNAi in the *H*. *glycines*-resistant *G*. *max*_[Peking/PI 548402]_. These experiments have been performed to determine if an engineered impairment of the normal defense response could be generated. The combination of engineered resistance in the *G*. *max*_[Williams 82/PI 518671]_ genotype and engineered susceptibility in the *G*. *max*_[Peking/PI 548402]_ genotype is indicative that the gene functions within the defense response to *H*. *glycines* parasitism [13]. The effect that the expression of the OE or RNAi transgene has on the relative transcript abundance of its target has been determined by qRT-PCR, confirming the target gene cassettes are functioning as expected as compared to the ribosomal S21 control (p < 0.05). The qRT-PCR experiments show that the candidate gene is increased in its relative expression in the overexpressing lines as compared to the pRAP15 control (p < 0.05) (**Fig 5**). The increase in target gene expression has a range of 3.23 fold for the secreted peroxidase (Glyma.01G171100) to 5.66 for the endomembrane protein 70 protein (Glyma.09G096700) (**Fig 5**). In contrast, the qRT-PCR experiments show that the candidate gene is decreased in its relative expression in the RNAi lines as compared to the pRAP17 control (p < 0.05) (**Fig 5**). The decrease of target gene expression has a range of -3.19 fold for the endomembrane protein 70 protein (Glyma.09G096700) to -4.99 for galactose mutarotase-like superfamily protein (Glyma.19G020700) (**Fig 5**).

### Functional analyses of the MAPK-All, syncytium-expressed genes having secretion signals

**Fig 6:**
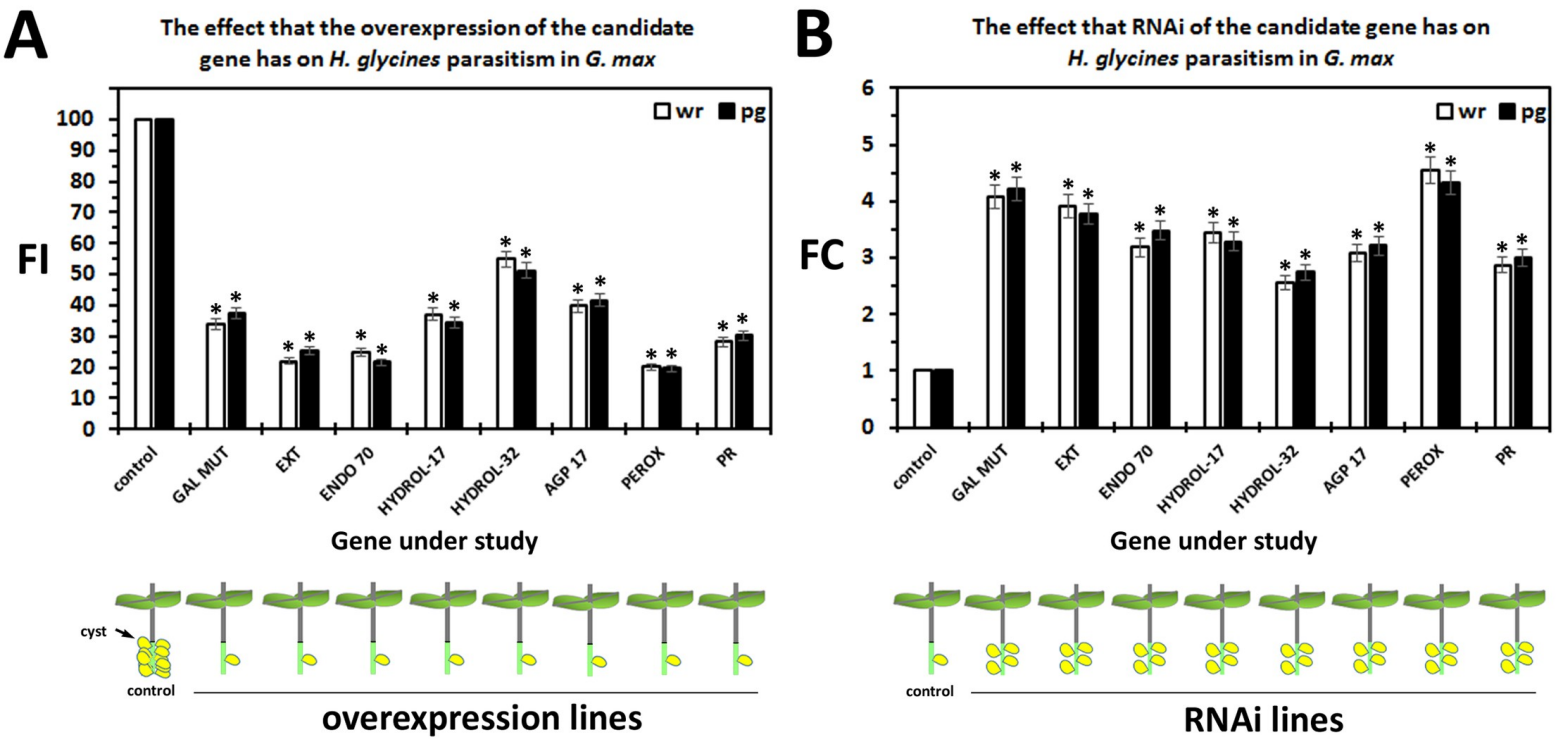
Functional transgenic analyses. The analyses demonstrate that the genetic engineering of the putatively secreted candidate defense gene functions as expected in the *G*. *max* defense to *H*. *glycines*. The calculated FI for the OE and RNAi lines is presented as compared to the controls. The analyzed genes are a galactose mutarotase-like (GAL MUT, Glyma.19G020700), Pollen Ole e 1 allergen and extensin family protein (EXT, Glyma.13G178700); endomembrane protein 70 protein family (ENDO 70, Glyma.09G096700); O-Glycosyl hydrolases family 17 protein (HYDROL-17, Glyma.14G020000); glycosyl hydrolases family 32 protein (HYDROL-32, Glyma.13G349300); FASCICLIN-like arabinogalactan protein 17 precursor (AGP 17, Glyma.12G096300); peroxidase superfamily protein (PEROX, Glyma.01G171100); pathogenesis-related thaumatin superfamily protein (PR, Glyma.12G064300). * Statistically significant, p < 0.05 calculated by the Mann–Whitney–Wilcoxon (MWW) Rank-Sum Test [35]. The experimental error representing standard deviation is presented. The results are the average of three independently run biological replicates, all p values < 0.001.

Experiments have been conducted to determine the effect that the expression of the transgene cassettes have on *H*. *glycines* parasitism. The roots have been infected with 2,000 J2s and infection has been allowed to proceed for 30 days. At the end of the infection period, the *H*. *glycines* cysts have been collected from the whole root and associated soil samples (wr) in which the transgenic plant had been growing. Furthermore, the roots have been weighed to permit the standardization of the cyst data to the mass of the root in cyst per gram (pg) of root. The pg analysis have been done to take into consideration whether or not the overexpression or RNAi of the transgene affects root development. The enumerated female index (FI) for each transgene has revealed that the overexpression of the candidate defense genes has led to a decrease in parasitism with a low of 19.56% observed in the pg analysis of the secreted peroxidase (Glyma.01G171100) to a high of 54.87% observed in the wr analysis of hydrolase-32 (Glyma.13G349300) (p < 0.001) (**Fig 6**). To obtain a clearer understanding of whether these genes play a defense role, RNAi of these same 8 candidate resistance genes has been performed in the *H*. *glycines*-resistant *G*. *max*_[Peking/PI 548402]_ to determine if engineered susceptibility could be obtained (**Fig 6**). The results show that *H*. *glycines* parasitism can be increased to statistically significant levels (p < 0.001). The largest increase in parasitism has been observed in the whole root analyses for the secreted peroxidase (Glyma.01G171100) (4.54 fold) while the smallest increase in parasitism has been observed for the hydrolase-32 gene (Glyma.13G349300) (2.56 fold). The combination of a statistically significant decrease in parasitism in the overexpression lines and statistically significant increase in parasitism in the RNAi lines is the criteria required for the gene have a role in defense in our analysis. The similarity in the values obtained for the cysts per whole root and cysts per gram of root indicates that there is no effect on root growth caused by the transgene.

## Discussion

The involvement of MAPKs in *G*. *max* biological processes include, but are not limited to defense to different pathogens, arbuscular mycorrhizal associations during drought tolerance, wound responses, growth and other activities [37–43]. The annotation of the MAPK gene family presented by McNeece et al. [11] had been based off of earlier studies that led to the identification of different numbers of MAPKs. These differences in gene count arose due to the analyses procedures used [39, 44, 45]. McNeece et al. [11] presented a specific set of criteria used to select the chosen *G*. *max* MAPKs, based off of comparisons made to MAPKs already identified in *A*. *thaliana* and those already identified in *G*. *max*. Different numbers of *G*. *max* MAPKs, therefore, are possible due to the different set parameters [39, 44, 45]. The importance of the analysis became evident when MAPKs that had not previously been associated with defense had been shown to work in that manner by suppressing *H*. *glycines* parasitism when overexpressed while, in contrast, facilitating parasitism when undergoing RNAi. The 9 MAPKs that have been identified as functioning during the defense response that *G*. *max* has to *H*. *glycines* are referred to as defense MAPKs [11]. However, many details regarding these MAPKs remained to be explored.

### A genetic pathway for MAPK signal transduction in relation to *G*. *max* defense to *H*. *glycines*

Prior analyses have demonstrated that the bacterial effector harpin is capable of inducing the expression of GmNDR1-1 (Glyma.12G214100) as well as specific MAPKs [11, 16, 17, 20]. Furthermore, harpin can induce the expression of a *G*. *max* BIK1 (GmBIK1) [20]. The experiments went on to demonstrate the overexpression of GmBIK1 (Glyma.14G068700) can lead to the induction of MAPK3-1 and MAPK3-2 expression [11]. The MAPKs have then been shown to influence the expression of other MAPKs, functioning in complex ways in the *G*. *max*-*H*. *glycines* pathosystem during defense [11]. In those studies, the MAPKs have been shown to influence the expression of a panel of proven defense genes including genes functioning in salicylic acid (SA) signaling. These *G*. *max* defense genes included ENHANCED DISEASE SUSCEPTIBILITY1 (EDS1) (Glyma.06G187200), NONEXPRESSOR of PR1 (NPR1) (Glyma.09G020800) and TGA2 (Glyma.10G296200) [46–48]. Other *G*. *max* defense genes included the ABC-G-type transporter (Glyma.17G039300). Furthermore, genes functioning in vesicle transport have been identified. These *G*. *max* defense genes included the *rhg1* component alpha soluble NSF attachment protein (Gmα-SNAP-5) (Glyma.18G022500), mammalian uncoordinated18 (GmMUNC18-5) (Glyma.11G029600) and synaptosomal-associated protein 25 (GmSNAP-25-3) (Glyma.04G164400). Other *G*. *max* defense genes included those with characteristics of secreted proteins including xyloglucan endotransglycosylase/hydrolase (GmXTH43) (Glyma.17G065100), reticuline oxidase (GmRO-40) (Glyma.15G132800), α-hydroxynitrile glucosidase (Gmβg-4) (Glyma.11G129600) and pathogenesis related 1–6 (PR1-6) (Glyma.15G062400) and genes involved in carbon metabolism serine hydroxymethyltransferase (GmSHMT-5) (Glyma.08G108900) and galactinol synthase (GmGS-3) (Glyma.19G227800) [11]. Those experiments have provided a framework for understanding how the GmMAPKs function in relation to ETI and PTI and the defense process to *H*. *glycines* [11].

The experiments presented here build on those studies by analyzing RNA seq data that has been obtained from the MAPK-OE lines for each of the 9 *G*. *max* defense MAPKs [28]. While Alshehri et al. [28] had developed a database to house and analyze the RNA seq data, the actual data had not been studied in a manner that would identify candidate defense genes that may be involved in impairing *H*. *glycines* parasitism. That analysis has been presented here. The analysis presented here has been able to put the MAPK RNA seq data into the perspective of previously identified gene expression events occurring specifically within the syncytium undergoing the process of defense in two different *G*. *max* genotypes that are capable of a defense response (*G*. *max*_[Peking/PI 548402]_ and *G*. *max*_[PI 88788]_) [26, 29]. By comparing the MAPK-all-OE RNA seq and syncytium expression data sets, it has been possible in the analysis presented here to obtain a perspective regarding the defense process that compliments the earlier work.

### RNA seq analyses identify a core gene set expressed in common between the defense MAPKs

The RNA seq analyses presented here have identified a core set of 309 genes that are increased in their relative transcript abundance while 815 are decreased among the MAPK-all-OE lines as compared to their control (p < 0.05). These results are consistent with gene expression studies performed in *A*. *thaliana* that have shown changes in gene expression occurring at the genomic level during a defense response [49]. Among the genes that are increased in their relative transcript abundance are a number of receptors having defense functions as resistance (R) genes. These genes will be described briefly as some of them relate to previously identified genes that function during the defense response that *G*. *max* has to *H*. *glycines*.

The *G*. *max* R genes that have been identified in the analysis presented here include leucine rich repeat (LRR) and nucleotide-binding adaptor shared by APAF-1, R proteins, and CED-4 domain (NB-ARC) -containing disease resistance proteins (Glyma.18G078000 [RESISTANCE TO PSEUDOMONAS SYRINGAE PV MACULICOLA1 (RPM1)]; Glyma.07G065600; Glyma.07G065000). RPM1 confers resistance to the bacterial pathogen *Phytopthora syringae strains carrying the* avrRpm1 protein [50]. RPM1 functions together as a unit with a membrane receptor NDR1. NDR1 binds three proteins, RPM1, RESISTANCE TO PSEUDOMONAS SYRINGAE2 (RPS2) and RPM1-INTERACTING PROTEIN 4 (RIN4) to work in various ways leading to the activation of MAPK signaling [19, 51–54]. Our earlier work has studied the role that *G*. *max* NDR1 has in its defense response to *H*. *glycines* showing it functions effectively in the defense process [11, 20, 55].

Other *G*. *max* R genes that have been identified include the coiled coil (CC) NB LRR (Glyma.19G198900 [NDR1]). NDR1 can be activated by the bacterial effector harpin [16–18, 56]. Harpin then transduces the signal through its interacting proteins. As stated previously, NDR1 functions through RPM1, RPS2 and RIN4, leading to the activation of the MAPK cascade to elicit a defense response [16–19, 53, 56–58].

The analysis presented here has identified other *G*. *max* R genes including *the* toll interleukin 1 receptor, nucleotide-binding site, leucine-rich repeat (TIR-NBS-LRR) class disease resistance proteins (Glyma.19G055000 [TMV resistance protein N]; Glyma.10G263200; Glyma.15G232400 [TMV resistance protein N]; Glyma.12G238600 [Resistant to *Phakopsora pachyrhizi* {RPP1}-like]). Tobacco mosaic virus (TMV) resistance protein N functions in resistance to the tobacco mosaic virus [59]. (RPP1) is a locus first identified in *G*. *max* in germplasm exhibiting resistance to the basidiomycete pathogen *Phakopsora pachyrhizi* [60]. RPP1 interacts with avirulence determinants from the oomycete *Peronospora parasitica* to mediate resistance to different strains [61]. A RPP1 homolog has since been cloned, functioning in resistance to *P*. *pachyrhizi* [62].

A number of LRR containing genes have been identified (Glyma.10G263200; Glyma.12G238600; Glyma.13G266300; Glyma.08G083300 [FLAGELLIN SENSING2 {FLS2}]; Glyma.10G195700 [DAMP PEPTIDE 1 RECEPTOR {PEPR1}]). The *A*. *thaliana* FLS2 protein has been shown to interact with a bacterial flagellin protein epitope to activate BRASSINOSTEROID INSENSITIVE1 ASSOCIATED KINASE1 (BAK1) [22, 25]. In turn, BAK1 phosphorylates BIK1 [28, 63, 64]. BIK1 can also be autophosphorylated, indicating that it functions differently than BAK1 [65]. Pant et al. [13] has demonstrated the function of the *G*. *max* BIK1 in defense in the *G*. *max*-*H*. *glycines* pathosystem. Furthermore, the *G*. *max* BIK1 results in an increase in GmMAPK3 transcription [11]. This class of genes also includes the *A*. *thaliana* AtPEPR1 which is shared between BIK1 and BAK1 [25]. Out of all of the receptors described here, only NDR1 has been shown to be expressed within the syncytium and induced by MAPK expression. The remaining receptors are only induced in their expression in the MAPK-OE-all.

### The identification of a core MAPK-induced defense transcriptome

A relatively low number of genes have been identified in the RNA seq screen presented here as compared to the *G*. *max* genome having 88,647 transcripts and 56,044 protein-coding loci. These results indicate that the transcriptional screen had been successful in terms of yielding a set of genes that would be manageable in functional studies such as those presented here. However, the genes then had been analyzed further to fulfill a narrower scope sought for the functional transgenic screens.

The 309 induced genes that have been identified in the MAPK-OE-all screen is relatively low and manageable for transgenic testing. However, we sought ways to further narrow down the gene list while also learning more about the properties of the genes that compose that group. We have been able to compare the 309 MAPK-OE-all induced genes to gene expression data generated from specific root cell types found in *G*. *max*_[Peking/PI 548402]_ and *G*. *max*_[PI 88788]_. Prior analyses of the syncytium undergoing the defense process has been done [26, 29]. The experiments resulted in the identification of a number of genes that are expressed specifically within the syncytium during the defense processes [26]. It had been expected here that a number of the 309 MAPK-OE-all induced genes would not be expressed at the site of the defense response. Such an outcome would further narrow down the quantity of genes. The result of the analyses on the 309 MAPK-OE-all induced genes left 167 genes that we were able to obtain gene expression data for. An analysis then had been performed to determine whether Affymetrix® probe sets for these genes measured gene expression within the pericycle cells prior to parasitism (control) and parasitized root cells at 3 and 6 dpi [26]. In an examination of these data, we identified 7 different types of gene expression patterns. The characteristics of the gene activity have been referred to as expression Types 1–7. The main point taken from this analysis is that there had been 71 genes whereby detectable gene expression could be made for at least 1 of the 3 *G*. *max* root cell types (pericycle, 3 or 6 dpi syncytium undergoing the defense response) cell types. We observed that 45 of these genes are not expressed within the 0 dpi control, but are subsequently expressed in some combination within the 3 and 6 dpi time point samples (Types 1–3). We then observed that 26 genes incorporate expression at the 0 dpi time point (Types 4–6). The remaining 97 genes did not have measurable expression according to our criteria in any of the cells at the 0, 3 or 6 dpi time points, but have been shown to be induced in the MAPK-all-OE induced RNA seq analyses.

### MAPK-induced genes expressed at 3 and 6 dpi syncytium-signal peptide predicted function in defense (Type 1)

**Fig 7:**
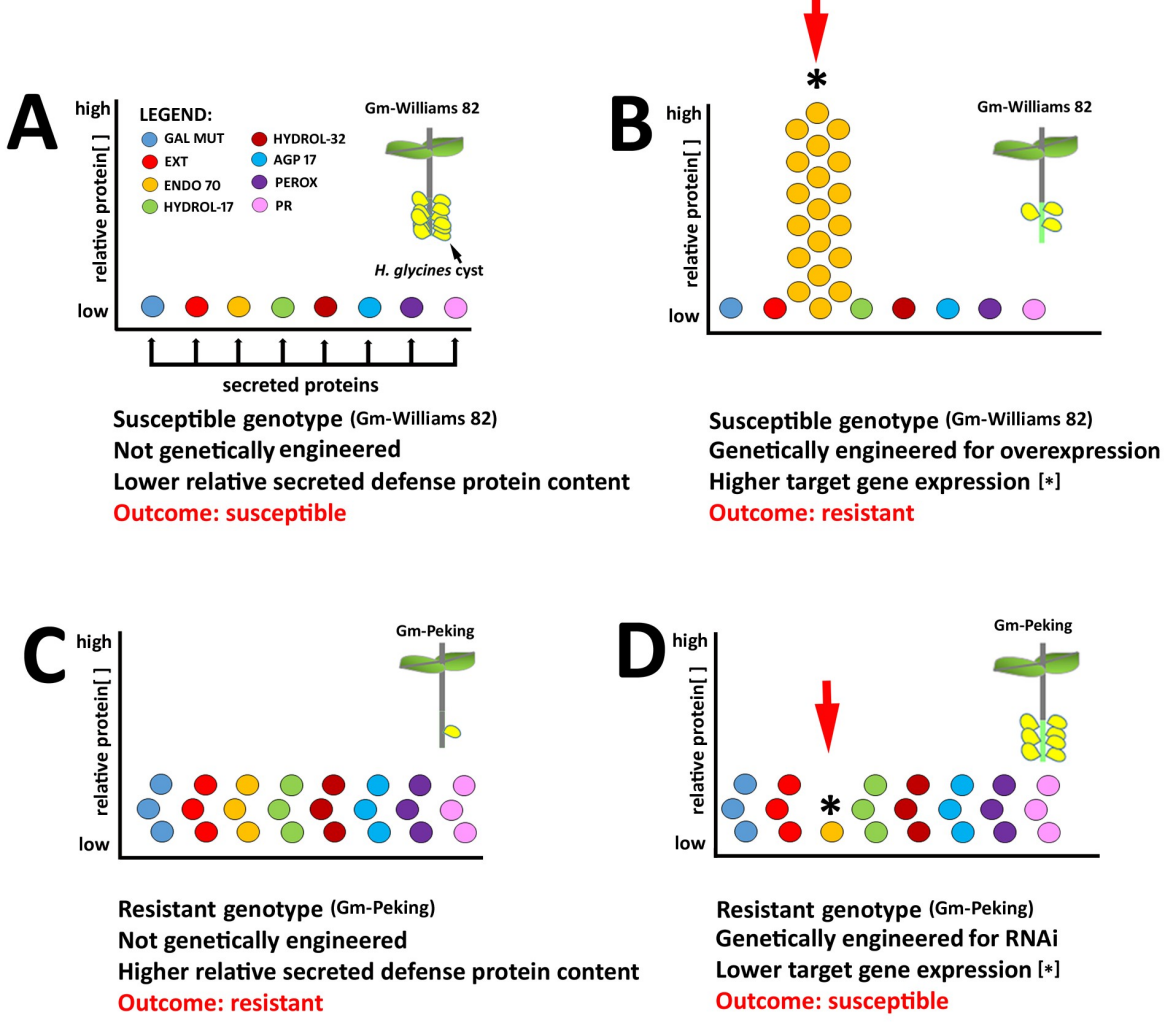
Model. **A.** Example of the relatively low expression concentration of 8 candidate resistance genes in a naturally *H*. *glycines*-susceptible genotype (i.e. *G*. *max*_[Williams 82/PI 518671]_) shown with 10 *H*. *glycines* cysts. **B.** The *H*. *glycines*-susceptible genotype (i.e. *G*. *max*_[Williams 82/PI 518671]_), when engineered to overexpress one of the 8 candidate defense genes, results in a resistant reaction. The reaction is not exactly like a naturally occurring resistant reaction like that occurring in *G*. *max*_[Peking/PI 548402]_ (like shown in C) as indicated by the presence of more cysts (n = 3) than the naturally occurring resistant reaction (n = 1, in C). The level of engineered resistance would depend on the potency and/or timing of the expression of the gene. **C.** 8 Candidate defense genes have been determined to be more highly expressed in the syncytium undergoing a defense reaction to *H*. *glycines* parasitism in a naturally *H*. *glycines*-resistant genotype (i.e. *G*. *max*_[Peking/PI 548402]_). **D.** The *H*. *glycines*-resistant genotype (i.e. *G*. *max*_[Peking/PI 548402]_), when engineered to undergo RNAi of one of the candidate defense genes, results in an engineered susceptible reaction. The reaction is not exactly like a naturally occurring susceptible reaction as found in *G*. *max*_[Williams 82/PI 518671]_ (as shown in A) as indicated by fewer cysts (n = 7) than the naturally occurring susceptible reaction (n = 10, in A). The level of engineered susceptibility would depend on the importance of the gene to the defense reaction, its potency and/or the timing of its expression. The relative numbers of cysts are arbitrary for descriptive purposes.

Our analysis has identified 16 genes that are not expressed at 0 dpi, but then are expressed at the 3 and 6 dpi time points. Five of these genes are predicted to have secretion signals and are described here. The *G*. *max* galactose mutarotase-like superfamily protein (Glyma.19G020700) has not been studied in plants. Therefore, the functional work obtained in the study presented here is new to plants. However, the *Saccharomyces cerevisiae* galactose mutarotase protein functions in maintaining the equilibrium between the α- and β-anomers of galactose [66]. Ultimately, d-galactose is metabolized to d-glucose 1-phosphate whereby it is converted to d-glucose 6-phosphate which can enter glycolysis [66]. Glucose 1-phosphate can also be bound to uridine triphosphate through the activities of UDP glucose pyrophosphorylase to produce UDP glucose. In *A*. *thaliana* UDP glucose phosphorylase functions in regulating programmed cell death and is essential [67, 68]. The *G*. *max* pollen Ole e 1 allergen and extensin family protein (arabinogalactan (AGP) (Glyma.13G178700) has been shown in *A*. *thaliana* to perform roles in root epidermal cell elongation [69]. Furthermore, the *A*. *thaliana* AGP mutation root epidermal bulger 1 (reb-1) results in altered cortical microtubules, indicating a cross talk between cell wall modifying proteins and the cytoskeleton [70]. The *G*. *max* endomembrane protein 70 protein (EMP70) family (Glyma.09G096700) is a phylogenetically conserved protein. Experiments that have isolated *A*. *thaliana* Golgi proteins have revealed that EMP70 represents 25% of the quantified Golgi proteome [71]. Furthermore, EMP70 represents 50% of the top 10 most abundant Golgi proteins, but has been stated to have limited functional characterization [71]. Pant et al. [13] have already demonstrated the functionality of a xyloglucan endrotransglycosylase hydrolase (XTH) during the defense response *G*. *max* has toward *H*. *glycines* parasitism, reducing infection by 90%. XTH is processed through the Golgi apparatus, revealing the importance of the Golgi apparatus to defense in the *G*. *max*-*H*. *glycines* pathosystem [72]. The O-glycosyl hydrolases family 17 protein (Glyma.14G020000) and glycosyl hydrolases family 32 protein (Glyma.13G349300) relate to recent experiments performed in *Nicotiana benthamiana* [73]. The *A*. *thaliana* O-glycosyl hydrolases family 17 has been shown to promote hydrolytic elicitor release and consequently acting in immunity against the pathogenic bacterium *Pseudomonas syringae* [73]. The analyses presented here have provided further support for the hypothesis that the secretion system of *G*. *max* performs an important role that leads to its ability to overcome *H*. *glycines* parasitism (**Fig 7**) [26]. The experiments have also identified genes having other types of expression (Types 2–7) that may represent a pool of candidate genes with important functions in the defense response that *G*. *max* has toward *H*. *glycines*.

### Downregulated genes

A number of downregulated genes have been identified during the course of the analyses. The identification of downregulated genes (as well as upregulated genes) is a common finding among any genomics-based analyses and it can represent how an event influences a number processes occurring within an organ. Early genomics studies have identified a shift from housekeeping to defense processes occurring within plant organs undergoing a defense process [49]. Consequently, identifying suppressed genes here is not surprising. The MAPK-based study presented here was only designed to identify genes that are induced or suppressed by certain MAPKs from RNA isolated from uninfected roots. The types of genes that have been identified may or may not have defense functions when examining the specific cells that are undergoing infection or even specific races/pathotypes of *H*. *glycines*. There also maybe specific defense functions for certain members of gene families like what has been shown in *G*. *max* for its MAPKs [11]. This would represent a shift from housekeeping functions to defense functions within these gene families. Further study is required to examine why some of these genes appear to be suppressed in common between the different defense MAPKs and is a rich area for future analysis.

## Supporting information

S1 Fig Prediction of signal peptides. SignalP 5.0 has been employed, using default settings to identify the likelihood of having a predicted signal peptide. There are three types of peptides that can be identified, including a Sec signal peptide (Sec/SPI), a Lipoprotein signal peptide (Sec/SPII), a Tat signal peptide (Tat/SPI). Furthermore, No signal peptide at all (Other) could be determined [36].
(TIF)

S1 Table PCR and qRT-PCR primers. (XLSX)

S2 Table The RNA seq data for the MAPK-OE-all experiment. **Footnote**: The RNA seq samples of Alshehri et al. [28] presented here had been obtained from roots overexpressing MAPK2, MK3-1, MAPK3-2, MAPK4-1, MAPK5-3; MAPK6-2; MAPK13-1; MAPK16-4, MAPK20-2. The MAPK-OE-all gene list has been derived from genes expressed in common between these samples. Cutoff is p < 0.05.
(XLSX)

S3 Table The 309 MAPK-OE-all induced genes. (XLSX)

S4 Table The 815 MAPK-OE-all suppressed genes. (XLSX)

S5 Table The 167 MAPK-OE-all genes that have Affymetrix^®^ probe set identifiers. (XLSX)

S6 Table The Type 7 genes identified in the analysis of the MAPK-OE-all genes that have Affymetrix^®^ probe set identifiers. (XLSX)

## References

[1] Sturgill TW, Ray LB. Muscle proteins related to microtubule associated protein-2 are substrates for an insulin-stimulatable kinase. Biochem Biophys Res Commun. 1986; 134(2): 565–571. 10.1016/s0006-291x(86)80457-0 3511906

[2] Han J, Lee JD, Bibbs L, Ulevitch RJ. A MAP kinase targeted by endotoxin and hyperosmolarity in mammalian cells. Science (80-). 1994; 265(5173): 808–811. 10.1126/science.7914033 7914033

[3] Suzuki K, Shinshi H. Transient activation and tyrosine phosphorylation of a protein kinase in tobacco cells treated with a fungal elicitor. Plant Cell. 1995; 7(5): 639–647. 10.1105/tpc.7.5.639 12242379 PMC160810

[4] Jonak C, Kiegerl S, Ligterink W, Barker PJ, Huskisson NS, Hirt H. Stress signaling in plants: A mitogen-activated protein kinase pathway is activated by cold and drought. Proc Natl Acad Sci. 1996; 93(20): 11274–11279. 10.1073/pnas.93.20.11274 8855346 PMC38320

[5] Jonak C, Ökrész L, Bögre L, Hirt H. Complexity, cross talk and integration of plant MAP kinase signalling. Vol. 5, Current Opinion in Plant Biology. 2002 p. 415–424. 10.1016/s1369-5266(02)00285-6 12183180

[6] Huang CYF, Ferrell JE. Ultrasensitivity in the mitogen-activated protein kinase cascade. Proc Natl Acad Sci. 1996; 93(19): 10078–10083. 10.1073/pnas.93.19.10078 8816754 PMC38339

[7] Ligterink W, Kroj T, Zur Nieden U, Hirt H, Scheel D. Receptor-mediated activation of a MAP kinase in pathogen defense of plants. Science (80-). 1997; 276(5321): 2054–2057. 10.1126/science.276.5321.2054 9197271

[8] Wrather JA, Koenning SR. Estimates of disease effects on soybean yields in the United States 2003 to 2005. J Nematol. 2006; 38(2): 173–180. 19259444 PMC2586459

[9] Ren D, Liu Y, Yang KY, Han L, Mao G, Glazebrook J, et al A fungal-responsive MAPK cascade regulates phytoalexin biosynthesis in Arabidopsis. Proc Natl Acad Sci U S A. 2008; 105(14): 5638–5643. 10.1073/pnas.0711301105 18378893 PMC2291085

[10] Miles GP, Samuel MA, Zhang Y, Ellis BE. RNA interference-based (RNAi) suppression of AtMPK6, an *Arabidopsis* mitogen-activated protein kinase, results in hypersensitivity to ozone and misregulation of AtMPK3. Environ Pollut. 2005; 138(2): 230–237. 10.1016/j.envpol.2005.04.017 15964670

[11] McNeece BT, Sharma K, Lawrence GW, Lawrence KS, Klink VP. The mitogen activated protein kinase (MAPK) gene family functions as a cohort during the *Glycine max* defense response to *Heterodera glycines*. Plant Physiol Biochem. 2019; 137: 25–41. 10.1016/j.plaphy.2019.01.018 30711881

[12] Ross J. Host-Parasite relationship of the soybean cyst nematode in resistant soybean roots. Phytopathology. 1958; 48: 578–579.

[13] Pant SR, Matsye PD, McNeece BT, Sharma K, Krishnavajhala A, Lawrence GW, et al Syntaxin 31 functions in *Glycine max* resistance to the plant parasitic nematode Heterodera glycines. Plant Mol Biol. 2014; 85(1–2): 107–121. 10.1007/s11103-014-0172-2 24452833

[14] Sharma K, Pant SR, McNeece BT, Lawrence GW, Klink VP. Co-regulation of the *Glycine max* soluble N-ethylmaleimide-sensitive fusion protein attachment protein receptor (SNARE)-containing regulon occurs during defense to a root pathogen. J Plant Interact. 2016; 11(1): 74–93.

[15] Jones JDG, Dangl JL. The plant immune system. Vol. 444, Nature. 2006 p. 323–329. 10.1038/nature05286 17108957

[16] Wei ZM, Laby RJ, Zumoff CH, Bauer DW, He SY, Collmer A, et al Harpin, elicitor of the hypersensitive response produced by the plant pathogen *Erwinia amylovora*. Science (80-). 1992; 257(5066): 85–88. 10.1126/science.1621099 1621099

[17] Gopalan S, Wei W, He SY. hrp gene-dependent induction of hin1: a plant gene activated rapidly by both harpins and the avrPto gene-mediated signal. Plant J. 1996; 10(4): 591–600. 10.1046/j.1365-313x.1996.10040591.x 8893538

[18] Century KS, Shapiro AD, Repetti PP, Dahlbeck D, Holub E, Staskawicz BJ. NDR1, a pathogen-induced component required for *Arabidopsis* disease resistance. Science (80-). 1997; 278(5345): 1963–1965. 10.1126/science.278.5345.1963 9395402

[19] Desikan R, Clarke A, Atherfold P, Hancock JT, Neill SJ. Harpin induces mitogen-activated protein kinase activity during defence responses in *Arabidopsis thaliana* suspension cultures. Planta. 1999; 210(1): 97–103. 10.1007/s004250050658 10592037

[20] Aljaafri WAR, McNeece BT, Lawaju BR, Sharma K, Niruala PM, Pant SR, et al A harpin elicitor induces the expression of a coiled-coil nucleotide binding leucine rich repeat (CC-NB-LRR) defense signaling gene and others functioning during defense to parasitic nematodes. Plant Physiol Biochem. 2017; 121: 161–175. 10.1016/j.plaphy.2017.10.004 29107936

[21] Veronese P, Nakagami H, Bluhm B, AbuQamar S, Chen X, Salmeron J, et al The membrane-anchored BOTRYTIS-INDUCED KINASE1 plays distinct roles in Arabidopsis resistance to necrotrophic and biotrophic pathogens. Plant Cell. 2006; 18(1): 257–273. 10.1105/tpc.105.035576 16339855 PMC1323497

[22] Li J, Chory J. A putative leucine-rich repeat receptor kinase involved in brassinosteroid signal transduction. Cell. 1997; 90(5): 929–938. 10.1016/s0092-8674(00)80357-8 9298904

[23] Chinchilla D, Zipfel C, Robatzek S, Kemmerling B, Nürnberger T, Jones JDG, et al A flagellin-induced complex of the receptor FLS2 and BAK1 initiates plant defence. Nature. 2007; 448(7152): 497–500. 10.1038/nature05999 17625569

[24] Zipfel C, Kunze G, Chinchilla D, Caniard A, Jones JDG, Boller T, et al Perception of the Bacterial PAMP EF-Tu by the Receptor EFR Restricts Agrobacterium-Mediated Transformation. Cell. 2006; 125(4): 749–760. 10.1016/j.cell.2006.03.037 16713565

[25] Liu Z, Wu Y, Yang F, Zhang Y, Chen S, Xie Q, et al BIK1 interacts with PEPRs to mediate ethylene-induced immunity. Proc Natl Acad Sci U S A. 2013; 110(15): 6205–6210. 10.1073/pnas.1215543110 23431184 PMC3625333

[26] Matsye PD, Kumar R, Hosseini P, Jones CM, Tremblay A, Alkharouf NW, et al Mapping cell fate decisions that occur during soybean defense responses. Plant Mol Biol. 2011; 77(4–5): 513–528. 10.1007/s11103-011-9828-3 21986905

[27] Zhang J, Lu H, Li X, Li Y, Cui H, Wen CK, et al Effector-triggered and pathogen-associated molecular pattern-triggered immunity differentially contribute to basal resistance to *Pseudomonas syringae*. Mol Plant-Microbe Interact. 2010; 23(7): 940–948. 10.1094/MPMI-23-7-0940 20521956

[28] Alshehri HA, Alkharouf NW, Darwish O, McNeece BT, Klink VP. MAPKDB: A MAP kinase database for signal transduction element identification. Bioinformation [Internet]. 2019; 15(5): 338–341. 10.6026/97320630015338 31249436 PMC6589469

[29] Klink V, Overall C, Alkharouf N, MacDonald M, Matthews B. Microarray detection calls as a means to compare transcripts expressed within syncytial cells isolated from incompatible and compatible soybean (*Glycine max*) roots infected by the soybean cyst nematode (*Heterodera glycines*). J Biomed Biotechnol. 2010; 1–30. 10.1155/2010/491217 PMC2875038 20508855

[30] Matsye PD, Lawrence GW, Youssef RM, Kim KH, Lawrence KS, Matthews BF, et al The expression of a naturally occurring, truncated allele of an α-SNAP gene suppresses plant parasitic nematode infection. Plant Mol Biol. 2012; 80(2): 131–155. 10.1007/s11103-012-9932-z 22689004

[31] Klink VP, Kim KH, Martins V, MacDonald MH, Beard HS, Alkharouf NW, et al A correlation between host-mediated expression of parasite genes as tandem inverted repeats and abrogation of development of female *Heterodera glycines* cyst formation during infection of *Glycine max*. Planta. 2009; 230(1): 53–71. 10.1007/s00425-009-0926-2 19347355

[32] Livak KJ, Schmittgen TD. Analysis of Relative Gene Expression Data Using Real-Time Quantitative PCR and the 2 C T Method. Methods. 2001; 25: 402–408. 10.1006/meth.2001.1262 11846609

[33] Yuan JS, Reed A, Chen F, Stewart CN. Statistical analysis of real-time PCR data. BMC Bioinformatics. 2006; 7(1): 1–12. 10.1186/1471-2105-7-85 16504059 PMC1395339

[34] Golden A, Epps J, Duclos L, Fox J, Bernard R. Terminology and identity of infraspecific forms of the soybean cyst nematode (*Heterodera glycines*). Plant Dis Reports. 1970; 54: 544–546.

[35] Mann HB, Whitney DR. On a Test of Whether one of Two Random Variables is Stochastically Larger than the Other. Vol. 18, The Annals of Mathematical Statistics. 1947 p. 50–60.

[36] Armenteros JJ, Tsirigos KD, Sønderby CK, Petersen TN, Winther O, Brunak S, et al SignalP 5.0 improves signal peptide predictions using deep neural networks. Nat Biotechnol. 2019; 37(4): 420–423. 10.1038/s41587-019-0036-z 30778233

[37] Lee S, Hirt H, Lee Y. Phosphatidic acid activates a wound-activated MAPK in *Glycine max*. Plant J. 2001; 26(5): 479–486. 10.1046/j.1365-313x.2001.01037.x 11439134

[38] Daxberger A, Nemak A, Mithöfer A, Fliegmann J, Ligterink W, Hirt H, et al Activation of members of a MAPK module in β-glucan elicitor-mediated non-host resistance of soybean. Planta. 2007; 225(6): 1559–1571. 10.1007/s00425-006-0442-6 17123101

[39] Liu JZ, Horstman HD, Braun E, Graham MA, Zhang C, Navarre D, et al Soybean homologs of MPK4 negatively regulate defense responses and positively regulate growth and development. Plant Physiol. 2011; 157(3): 1363–1378. 10.1104/pp.111.185686 21878550 PMC3252160

[40] Liu JZ, Braun E, Qiu WL, Shi YF, Marcelino-Guimarães FC, Navarre D, et al Positive and negative roles for soybean MPK6 in regulating defense responses. Mol Plant-Microbe Interact. 2014; 27(8): 824–834. 10.1094/MPMI-11-13-0350-R 24762222

[41] Liu Z, Li Y, Ma L, Wei H, Zhang J, He X, et al Coordinated regulation of arbuscular mycorrhizal fungi and soybean MAPK pathway genes improved mycorrhizal soybean drought tolerance. Mol Plant-Microbe Interact. 2015; 28(4): 408–419. 10.1094/MPMI-09-14-0251-R 25390189

[42] Xu HY, Zhang C, Li ZC, Wang ZR, Jiang XX, Shi YF, et al The MAPK kinase kinase GMMEKK1 regulates cell death and defense responses. Plant Physiol. 2018; 178(2): 907–922. 10.1104/pp.18.00903 30158117 PMC6181047

[43] Tian SN, Liu DD, Zhong CL, Xu HY, Yang S, Fang Y, et al Silencing GmFLS2 enhances the susceptibility of soybean to bacterial pathogen through attenuating the activation of GmMAPK signaling pathway. Plant Sci. 2020; 292: 110386 10.1016/j.plantsci.2019.110386 32005391

[44] Neupane A, Nepal MP, Piya S, Subramanian S, Rohila JS, Reese RN, et al Identification, Nomenclature, and Evolutionary Relationships of Mitogen-Activated Protein Kinase (MAPK) Genes in Soybean. Evol Bioinforma. 2013; 9(9): 363–386. 10.4137/EBO.S12526 PMC3785387 24137047

[45] Mohanta TK, Arora PK, Mohanta N, Parida P, Bae H. Identification of new members of the MAPK gene family in plants shows diverse conserved domains and novel activation loop variants. BMC Genomics. 2015; 16(1): 58 10.1186/s12864-015-1244-7 25888265 PMC4363184

[46] Hui Cao, Bowling SA Gordon AS, Xinnian Dong. Characterization of an *Arabidopsis* mutant that is nonresponsive to inducers of systemic acquired resistance. Plant Cell. 1994; 6(11): 1583–1592. 10.1105/tpc.6.11.1583 12244227 PMC160545

[47] Falk A, Feys BJ, Frost LN, Jones JDG, Daniels MJ, Parker JE. EDS1, an essential component of R gene-mediated disease resistance in *Arabidopsis* has homology to eukaryotic lipases. Proc Natl Acad Sci. 1999; 96(6): 3292–3297. 10.1073/pnas.96.6.3292 10077677 PMC15935

[48] Niggeweg R, Thurow C, Kegler C, Gatz C. Tobacco transcription factor TGA2.2 is the main component of as-1- binding factor ASF-1 and is involved in salicylic acid- and auxin-inducible expression of as-1-containing target promoters. J Biol Chem. 2000; 275(26): 19897–19905. 10.1074/jbc.M909267199 10751419

[49] Scheideler M, Schlaich NL, Fellenberg K, Beissbarth T, Hauser NC, Vingron M, et al Monitoring the switch from housekeeping to pathogen defense metabolism in *Arabidopsis thaliana* using cDNA arrays. J Biol Chem. 2002; 277(12): 10555–10561. 10.1074/jbc.M104863200 11748215

[50] Boyes DC, Nam J, Dangl JL. The Arabidopsis thaliana RPM1 disease resistance gene product is a peripheral plasma membrane protein that is degraded coincident with the hypersensitive response. Proc Natl Acad Sci. 1998; 95(26): 15849–15854. 10.1073/pnas.95.26.15849 9861059 PMC28133

[51] Mindrinos M, Katagiri F, Yu G-L, Ausubei FM. The A. thaliana Disease Resistance Gene RPS2 Encodes a Protein Containing a Nucleotide-Binding Site and Leucine-Rich Repeats. Cell. 1994; 78: 1089–1099. 10.1016/0092-8674(94)90282-8 7923358

[52] Grant MR, Godiard L, Straube E, Ashfield T, Lewald J, Sattler A, et al Structure of the *Arabidopsis* RPM1 gene enabling dual specificity disease resistance. Science (80-). 1995; 269(5225): 843–846. 10.1126/science.7638602 7638602

[53] Desikan R, Hancock JT, Ichimura K, Shinozaki K, Neill SJ. Harpin induces activation of the *Arabidopsis* mitogen-activated protein kinases AtMPK4 and AtMPK6. Plant Physiol. 2001; 126(4): 1579–1587. 10.1104/pp.126.4.1579 11500556 PMC117157

[54] Mackey D, Holt BF, Wiig A, Dangl JL. RIN4 interacts with *Pseudomonas syringae* type III effector molecules and is required for RPM1-mediated resistance in *Arabidopsis*. Cell. 2002; 108(6): 743–754. 10.1016/s0092-8674(02)00661-x 11955429

[55] McNeece BT, Pant SR, Sharma K, Niruala P, Lawrence GW, Klink VP. A Glycine max homolog of NON-RACE SPECIFIC DISEASE RESISTANCE 1 (NDR1) alters defense gene expression while functioning during a resistance response to different root pathogens in different genetic backgrounds. Plant Physiol Biochem. 2017; 114: 60–71. 10.1016/j.plaphy.2017.02.022 28273511

[56] Century KS, Holub EB, Staskawicz BJ. NDR1, a locus of *Arabidopsis thaliana* that is required for disease resistance to both a bacterial and a fungal pathogen. Proc Natl Acad Sci. 1995; 92(14): 6597–6601. 10.1073/pnas.92.14.6597 11607554 PMC41565

[57] Desikan R, Reynolds A, Hancock JT, Neill SJ. Harpin and hydrogen peroxide both initiate programmed cell death but have differential effects on defence gene expression in *Arabidopsis* suspension cultures. Biochem J. 1998; 330(1): 115–120. 10.1042/bj3300115 9461499 PMC1219116

[58] Lee J, Klessig DF, Nürnberger T. A harpin binding site in tobacco plasma membranes mediates activation of the pathogenesis-related gene HIN1 independent of extracellular calcium but dependent on mitogen-activated protein kinase activity. Plant Cell. 2001; 13(5): 1079–1093. 10.1105/tpc.13.5.1079 11340183 PMC135567

[59] Whitham S, Dinesh-Kumar SP, Choi D, Hehl C, Corr B, Baker B. The product of the tobacco mosaic virus resistance of gene N: similarity to teh toll fand the interleukic-1 receptor. Cell. 1994; 78: 1101–1115. 10.1016/0092-8674(94)90283-6 7923359

[60] Bromfield K, Hartwig E. Resistance to soybean rust and mode of inheritance. Crop Sci. 1980; 20: 254–255.

[61] Botella MA, Parker JE, Frost LN, Bittner-Eddy PD, Beynon JL, Daniels MJ, et al Three genes of the arabidopsis RPP1 complex resistance locus recognize distinct *Peronospora parasitica* avirulence determinants. Plant Cell. 1998; 10(11): 1847–1860. 10.1105/tpc.10.11.1847 9811793 PMC143951

[62] Pedley KF, Pandey AK, Ruck A, Lincoln LM, Whitham SA, Graham MA. Rpp1 encodes a ULP1-NBS-LRR protein that controls immunity to *Phakopsora pachyrhizi* in soybean. Mol Plant-Microbe Interact. 2019; 32(1): 120–133. 10.1094/MPMI-07-18-0198-FI 30303765

[63] Lu D, Wu S, Gao X, Zhang Y, Shan L, He P. A receptor-like cytoplasmic kinase, BIK1, associates with a flagellin receptor complex to initiate plant innate immunity. Proc Natl Acad Sci. 2010; 107(1): 496–501. 10.1073/pnas.0909705107 20018686 PMC2806711

[64] Lu D, Wu S, He P, Shan L. Phosphorylation of receptor-like cytoplasmic kinases by bacterial Flagellin. Plant Signal Behav. 2010; 5(5): 598–600. 10.4161/psb.11500 20404519 PMC3974576

[65] Lin W, Li B, Lu D, Chen S, Zhu N, He P, et al Tyrosine phosphorylation of protein kinase complex BAK1/BIK1 mediates *Arabidopsis* innate immunity. Proc Natl Acad Sci. 2014; 111(9): 3632–3637. 10.1073/pnas.1318817111 24532660 PMC3948311

[66] Scott A, Timson DJ. Characterization of the *Saccharomyces cerevisiae* galactose mutarotase/UDP-galactose 4-epimerase protein, Gal10p. FEMS Yeast Res. 2007; 7(3): 366–371. 17253981 10.1111/j.1567-1364.2006.00204.x

[67] Meng M, Geisler M, Johansson H, Harholt J, Scheller H V., Mellerowicz EJ, et al UDP-glucose pyrophosphorylase (UGPase) produces UDP. Plant Cell Physiol. 2009; 50(5): 998–1011. 10.1093/pcp/pcp052 19366709

[68] Chivasa S, Tomé DFA, Slabas AR. UDP-glucose pyrophosphorylase is a novel plant cell death regulator. J Proteome Res. 2013; 12(4): 1743–1753. 10.1021/pr3010887 23438466

[69] Ding L, Zhu JK. A role for arabinogalactan-proteins in root epidermal cell expansion. Planta. 1997; 203(3): 289–294. 10.1007/s004250050194 9431677

[70] Andème-Onzighi C, Sivaguru M, Judy-March J, Baskin TI, Driouich A. The reb1-1 mutation of *Arabidopsis* alters the morphology of trichoblasts, the expression of arabinogalactan-proteins and the organization of cortical microtubules. Planta. 2002; 215(6): 949–958. 10.1007/s00425-002-0836-z 12355155

[71] Nikolovski N, Shliaha P V., Gatto L, Dupree P, Lilley KS. Label-free protein quantification for plant golgi protein localization and abundance. Plant Physiol. 2014; 166(2): 1033–1043. 10.1104/pp.114.245589 25122472 PMC4213074

[72] Yokoyama R, Nishitani K. Endoxyloglucan Transferase is Localized both in the Cell Plate and in the Secretory Pathway Destined for the Apoplast in Tobacco Cells. Plant Cell Physiol. 2001; 42(3): 292–300. 10.1093/pcp/pce034 11266580

[73] Buscaill P, Chandrasekar B, Sanguankiattichai N, Kourelis J, Kaschani F, Thomas EL, et al Glycosidase and glycan polymorphism control hydrolytic release of immunogenic flagellin peptides. Science (80-). 2019; 364: 6436 10.1126/science.aav0748 30975858

