# Supplementary figures and images for: Mitogen activated protein kinase (MAPK)-regulated genes with predicted signal peptides function in the Glycine max defense response to the root pathogenic nematode Heterodera glycines
Source: PLoS One. 2020 Nov 4;15(11):e0241678. doi: 10.1371/journal.pone.0241678 (PMC7641413; doi:10.1371/journal.pone.0241678)

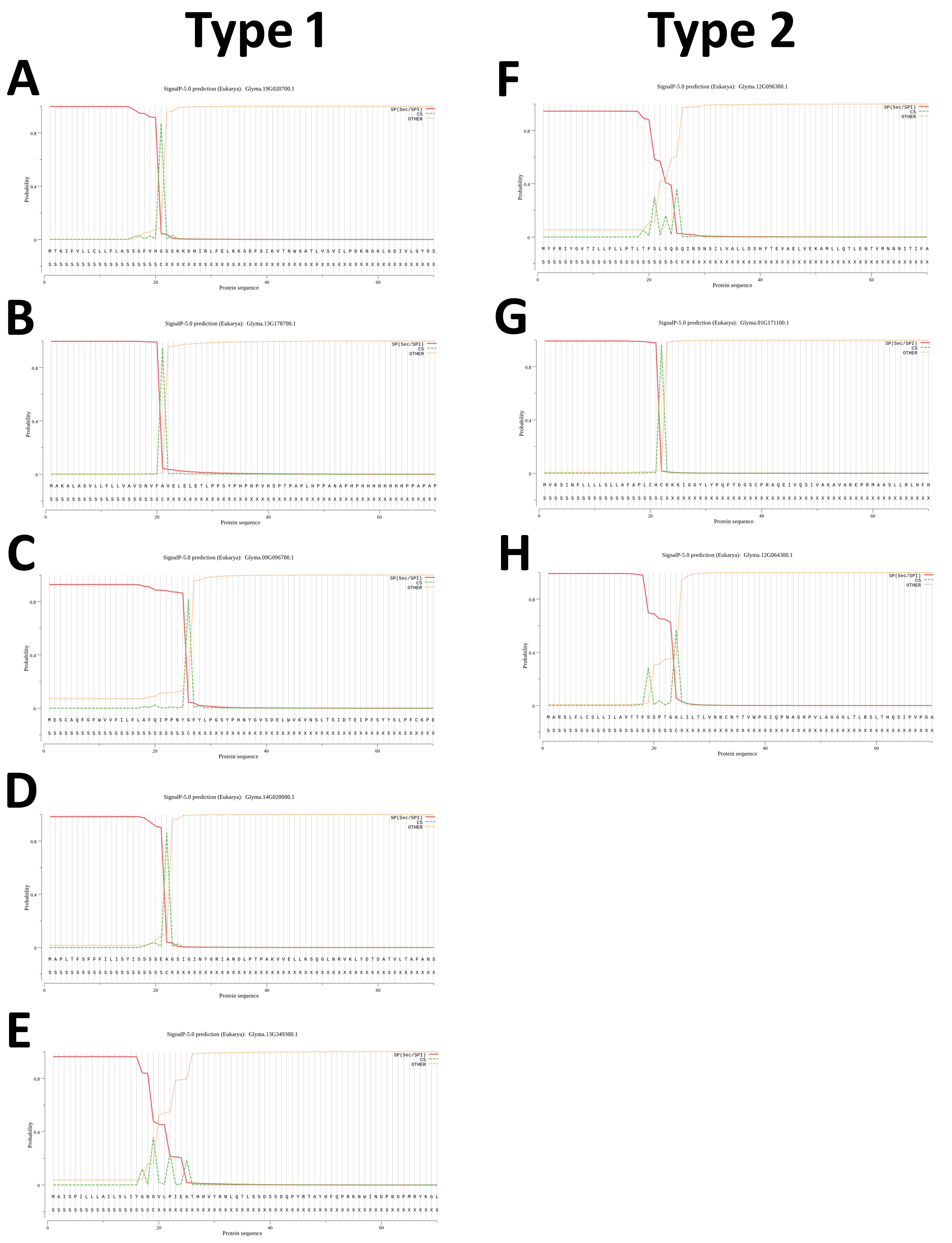

Supplement: S1 Fig — SignalP 5.0 has been employed, using default settings to identify the likelihood of having a predicted signal peptide. There are three types of peptides that can be identified, including a Sec signal peptide (Sec/SPI), a Lipoprotein signal peptide (Sec/SPII), a Tat signal peptide (Tat/SPI). Furthermore, No signal peptide at all (Other) could be determined [36]. (TIF) [file pone.0241678.s001.tif]
